# Synergistic Regulation of Alzheimer's Disease and Intestinal Microbiota Metabolism Mediated by the Gut-Brain Axis: A Comprehensive Analysis from a Multidisciplinary Perspective

**DOI:** 10.7150/ijms.135274

**Published:** 2026-07-30

**Authors:** Peng Lu, Maolin Liu, Lei Zhang, Jing-Jing Fan, Yan Sun

**Affiliations:** 1 Department of Clinical Laboratory, Cangzhou Central Hospital, Cangzhou, China.; 2 Department of Emergency ICU, Cangzhou Central Hospital, Cangzhou, China.; 3 Beijing Academy of Agriculture and Forestry Sciences, Beijing, China.

**Keywords:** gut-brain axis, Alzheimer's disease, gut microbiota, microbial metabolites, neuroinflammation, multidisciplinary integration, future perspectives

## Abstract

Alzheimer's disease (AD), as a neurodegenerative disease with the interaction of multiple factors, has a close association between its pathological process and the metabolic imbalance of the gut microbiota mediated by the gut-brain axis. This review systematically summarizes the molecular mechanisms by which the gut microbiota regulates the functions of the central nervous system bidirectionally through molecular pathways such as metabolites (e.g., short-chain fatty acids, tryptophan-kynurenine metabolites), immunomodulatory mediators (e.g., cytokines, chemokines), and bioactive substances (e.g., γ-aminobutyric acid, 5-hydroxytryptophan) via the gut-brain axis. It synthesizes current evidence suggesting the imbalance of microbiota homeostasis may be closely associated with the core pathologies of AD (including β-amyloid deposition and tau protein hyperphosphorylation) through mechanisms such as the activation of the C/EBPβ-AEP signaling pathway, induction of chronic neuroinflammation, oxidative stress cascade reactions, and metabolic network remodeling. These findings, primarily derived from preclinical models and correlational human studies, indicate potential mechanisms but require further causal validation and rigorous clinical translation, including the downregulation of butyrate synthesis pathways and their associated epigenetic and immunomodulatory consequences (as mechanistically dissected in Section 5.2). Multi-omics integration (metagenomics, metabolomics, spatial transcriptomics) has delineated characteristic microbial and metabolic alterations in AD, while computational approaches are beginning to elucidate the complex networks underlying these associations (see Sections 6 and 7 for details).Intervention strategies based on microbiota regulation (such as microbiota-targeted dietary interventions and postbiotics) are emerging as promising approaches, although their clinical applications remain in early stages. Preliminary evidence suggests that fecal microbiota transplantation may improve cognitive outcomes in AD patients with comorbid conditions; however, rigorous randomized controlled trials are essential to validate its efficacy and safety. Critically, translating these mechanistic insights into clinical practice requires overcoming three translational bottlenecks: inferring causality from correlational multi-omics data, resolving species/strain-level functional heterogeneity masked by genus-level taxonomy, and establishing standardized safety protocols for live biotherapeutic products. Addressing these challenges defines the near-term roadmap for precision medicine in AD. However, current research still faces challenges such as the heterogeneity of cross-omics data, the lack of technical standardization, and insufficient interdisciplinary cooperation mechanisms. In the future, it is necessary to promote the early molecular diagnosis and personalized targeted treatment of AD through longitudinal multi-omics dynamic monitoring, modeling of the microbiota-host interaction network, and optimization of the ethical-translational medicine framework.

## 1. Clinical and Molecular Characteristics of AD

AD, as a representative disease of degenerative diseases of the central nervous system, has clinical characteristics manifested as progressive cognitive impairment accompanied by abnormal mental behaviors. Its core neuropathological changes include senile plaques formed by the deposition of β-amyloid protein (Aβ), neurofibrillary tangles (NFTs) mediated by hyperphosphorylation of tau protein, neuroinflammatory responses associated with microglial cell activation, and mitochondrial dynamic imbalance^1^,^2^. At the molecular level, the pathogenesis of AD involves multiple pathophysiological processes such as oxidative stress cascade reactions, dysfunction of the cholinergic system, impairment of synaptic plasticity, and disruption of the integrity of the blood-brain barrier^3^,^4^. It is worth noting that recent research evidence indicates that the gut microbiota , through the bidirectional regulatory network of the gut-brain axis, may participate in the pathological evolution of AD through multiple molecular pathways^1^,^5^,^6^.In this review, we use “gut microbiota” as the primary term for the intestinal microbial community, while noting that “gut microbiome” and “intestinal microbiota” are frequently used interchangeably in the broader literature.

## 2. Biological Significance of the Gut-Brain Axis and Gut microbiota Metabolism

The gut-brain axis, as an important biological bridge connecting the intestinal ecosystem and the central nervous system, realizes cross-organ communication through pathways such as neuroendocrine, immunomodulation, and metabolic signal transduction^5^,^7^. gut microbiota affects nerve functions through molecular carriers such as metabolites (such as short-chain fatty acids, secondary bile acids, and tryptophan metabolites), immunomodulatory mediators (such as IL-6, TNF-α), and bioactive substances (such as γ-aminobutyric acid, 5-hydroxytryptamine)^7^-^9^. Clinical observations have found that there is a disorder in the structure of the gut microbiota(dysbiosis) in AD patients, manifested as a decrease in the relative abundance of Bacteroidetes, and a significant increase in the proportions of Firmicutes and Proteobacteria^10^,^11^. Among them, the abundance of the genus Clostridium is significantly positively correlated with the Aβ load in the brain^11^,^12^.

A critical barrier to clinical translation is that conventional 16S rRNA-based taxonomic profiling, while informative, conflates functionally divergent species and strains, yielding apparently contradictory literature that obscures true host-microbe interaction mechanisms. However, it is important to exercise caution when interpreting phylum-level compositional changes, as the Bacteroidetes phylum encompasses multiple genera(including Bacteroides, Prevotella, Alistipes, and Parabacteroides)each with distinct metabolic capabilities and immunomodulatory properties^13^,^14^. A systematic review of clinical studies found that findings regarding Bacteroidetes abundance in AD are mixed: some studies report decreased Bacteroides abundance in mild cognitive impairment (MCI) or AD patients, while others report increased abundance^10^,^15^. Even within studies focusing exclusively on the genus Bacteroides, contradictory results have been documented, with some showing AD-associated increases and others showing decreases depending on disease stage, APOE ε4 carrier status, and geographical cohort characteristics^16^,^17^. Furthermore, a recent reanalysis of 16S rRNA sequencing data using amplicon sequence variant (ASV) methods, which provide single-nucleotide resolution and are reproducible across studies, found no consistent phylum-level compositional pattern that distinguishes AD patients from controls, revealing substantial inter-individual heterogeneity in gut microbiota composition at all taxonomic levels^13^. These observations collectively indicate that the widely reported Firmicutes-to-Bacteroidetes ratio shift in AD should not be interpreted as a uniform or universally applicable diagnostic signature, and that species- and strain-level functional profiling (rather than phylum-level taxonomy alone) is essential for deciphering the causal relationships between gut microbiota dysbiosis and AD pathology^14^,^15^.

It is important to note, however, that the genus Clostridium exhibits substantial functional heterogeneity at the species and strain levels, which is often masked by genus-level taxonomic aggregation in conventional 16S rRNA-based analyses^13^,^14^. While some clinical correlational studies have reported a positive association between the abundance of Clostridium (referred to as an undifferentiated genus) and cerebral Aβ load, experimental evidence indicates that only specific Clostridium species and strains exert pathogenic or protective functions^18^,^19^. On the one hand, Clostridium perfringens (a Gram-positive, spore-forming anaerobic bacterium) produces epsilon toxin (ETX), one of the most potent bacterial toxins known. Intravenous or intracerebroventricular administration of sub-lethal concentrations of ETX induces permanent neuronal degeneration in the cortex, hippocampus, striatum and hypothalamus of rats and mice, accompanied by neurofilament accumulation, demyelination, synaptic stripping, and persistent behavioral impairments in surviving animals^9^,^19^. On the other hand, Clostridium butyricum, particularly the MIYAIRI 588 (CBM588) strain, has demonstrated unequivocal neuroprotective effects in APP/PS1 mouse models of AD^18^. CBM588 administration improves cognitive performance, alleviates colonic pathology through upregulation of tight junction proteins (Claudin 1, ZO-1, Occludin), reduces serum and brain LPS levels, decreases Aβ plaque deposition, and inhibits tau hyperphosphorylation via the JNK/CDK5/GSK-3β pathway^18^,^20^. The beneficial effects are mediated in part by increased production of (SCFAs) (particularly acetate) and modulation of gut microbiota composition without altering the overall flora structure^18^,^21^. Clostridium butyricum and its metabolite butyrate also significantly downregulate pro-inflammatory cytokines (IL-6, TNF-α) and upregulate anti-inflammatory IL-10, accompanied by increased abundance of butyrate-producing bacteria (e.g., Ruminococcus) and decreased abundance of pro-inflammatory genera^18^,^22^. Moreover, within the Clostridiaceae family, butyrate-producing clusters (Clostridium clusters IV and XIVa) are generally depleted in AD patients, whereas potentially pathogenic Clostridium species may undergo selective expansion under pathological conditions^23^,^24^. These findings underscore that the pathogenic or protective consequences of Clostridium colonization in AD are determined by specific species- and strain-level attributes (e.g., toxin production capacity, SCFA biosynthesis potential) and cannot be inferred from genus-level abundance changes alone^13^,^14^.

This dysbiosis of the microbiota may lead to abnormal permeability of the intestinal mucosal barrier, prompting pro-inflammatory factors such as lipopolysaccharide (LPS) to enter the systemic circulation through the “intestinal leakage” effect-a key trigger for peripheral and central inflammation (the downstream signaling cascade is explored in Section 5.2)^1^,^25^,^26^. The mechanistic roles of key pathways (including SCFAs-mediated epigenetic regulation, tryptophan-kynurenine metabolic shift, C/EBPβ-AEP signaling axis, and LPS-TLR4/NF-κB inflammatory cascade) will be systematically elaborated in Section 5.2.

## 3. Necessity of Interdisciplinary Research

**Figure 1:**
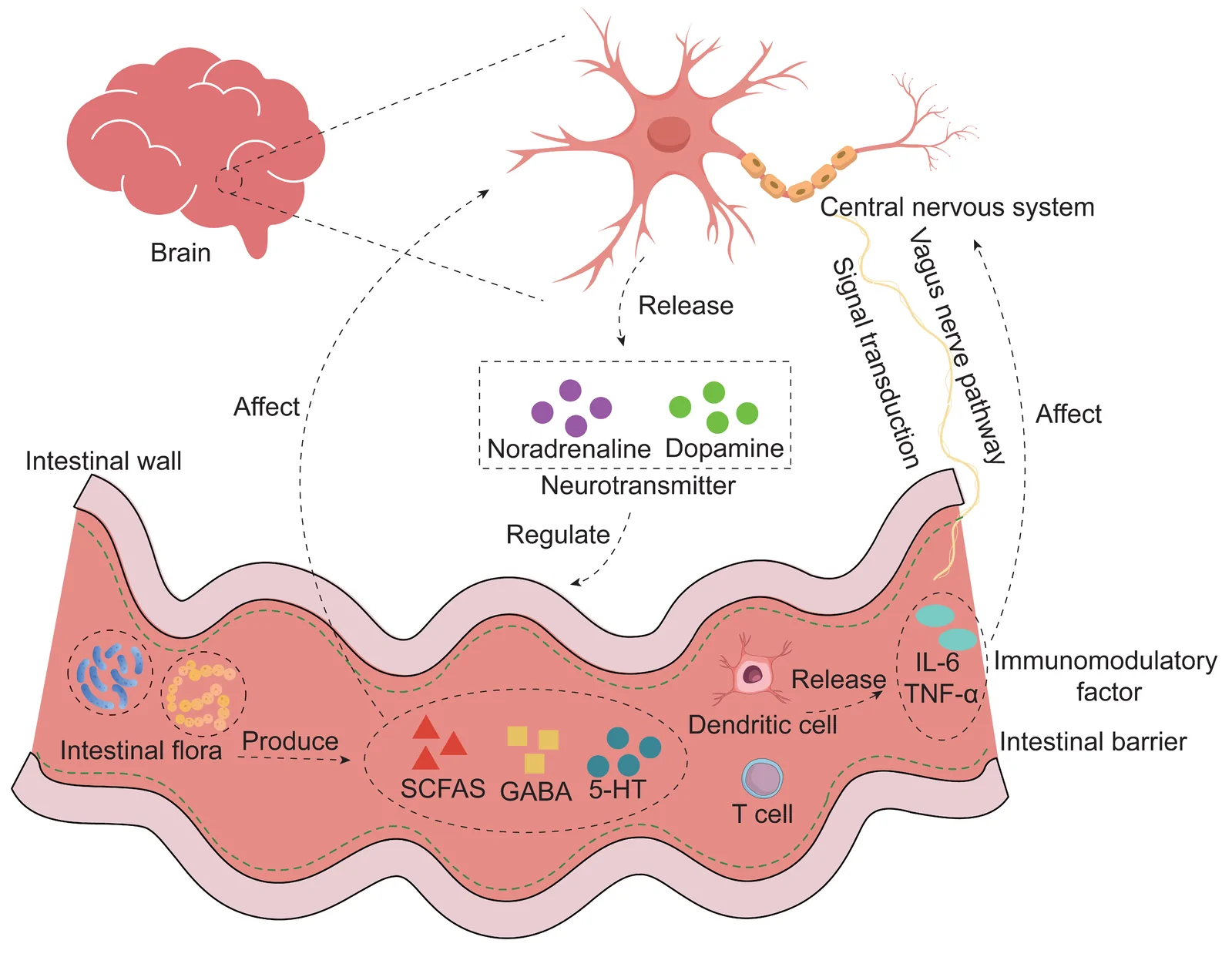
Interaction mechanism between gut microbiota and central nervous system. This figure illustrates the complex interaction mechanism between the gut microbiota and the central nervous system (CNS). Gut microbes (such as Bifidobacterium, Lactobacillus, etc.) influence intestinal barrier integrity, immune cell activation, cytokine release (e.g., IL-6, TNF-α), and neurotransmitter release/transmission by producing various metabolites (e.g., short-chain fatty acids [SCFAs], 5-hydroxytryptamine [5-HT], γ-aminobutyric acid [GABA]). Stimulated by gut microbes, immune cells release cytokines that enter the bloodstream, cross the blood-brain barrier, and affect the CNS, triggering inflammatory responses. Meanwhile, the gut microbiota connects to the CNS via the vagus nerve pathway, transmitting signals to regulate neural functions. The CNS, in turn, feedback-regulates the composition and functions of the gut microbiota by releasing neurotransmitters and other substances, thereby maintaining gut microenvironmental stability. This bidirectional regulatory mechanism plays a critical role in maintaining systemic homeostasis and is closely associated with the occurrence and development of multiple diseases.

Aiming at the complex pathological network of AD, the establishment of an interdisciplinary research system has important scientific value^3^,^27^. Integrating multi-omics technologies such as metagenomics, metabolomics, and proteomics can systematically analyze the key nodes of the metabolic interaction between the microbiota and the host^28^,^29^. For example, transplanting the fecal microbiota of AD patients into germ-free mice can induce an increase in Aβ deposition and spatial memory impairment in the recipient animals, which provides direct evidence for establishing the causal relationship between the microbiota and AD^30^,^31^. In terms of the development of intervention strategies, specific probiotics (such as Bifidobacterium) and metabolites (such as butyrate) have shown the potential to improve neuroinflammation and cognitive function in model animals, but their molecular mechanisms still need to be deeply elucidated through means of neuroimmunology and behavioral pharmacology^32^,^33^. The current research challenge lies in how to translate the basic research results into clinical applications^15^,^34^. Integrating biomarkers of the gut microbiota with the ATN (Aβ/tau/neurodegeneration) diagnostic system through artificial intelligence algorithms may improve the accuracy of early identification of AD^27^,^35^. In addition, personalized treatment programs based on microbiota regulation (including targeted dietary interventions and microbiota transplantation) need to verify their effectiveness and safety through multicenter clinical trials^32^,^36^. In conclusion, the research on the gut-brain axis has opened up a new dimension for exploring the mechanisms of AD, and the interdisciplinary integration will become the core driving force for analyzing the mechanisms of microbiota-neural interaction and developing precise diagnosis and treatment strategies^3^,^37^,^38^ (Fig. 1).

## 4. Critical Appraisal of Human Clinical Evidence: Findings, Limitations, and Translational Hurdles

While animal models have furnished pivotal mechanistic insights into gut-brain interactions in AD, the direct translation of these findings to human pathophysiology necessitates rigorous validation through clinical studies^2^,^15^. Current human evidence, though accumulating, presents a complex landscape marked by both promising associative findings and significant methodological challenges^3^,^39^.

### 4.1 Cross-sectional and longitudinal human studies

Cross-sectional studies have consistently demonstrated that patients with AD exhibit distinct gut microbiota compositional alterations compared to age-matched healthy controls^10^,^11^,^40^. A multi-cohort analysis revealed a consistent reduction in microbial α-diversity and a shift in β-diversity, characterized by decreased abundance of anti-inflammatory butyrate-producing genera (e.g., Faecalibacterium, Roseburia, Eubacterium) and increased abundance of pro-inflammatory taxa (e.g., Bacteroides, Alistipes)^17^,^41^. Specifically, Ferreiro et al. (2023) reported that gut microbiota composition alone could distinguish individuals with preclinical AD from cognitively normal controls with moderate accuracy (AUC = 0.68), suggesting that microbiota-derived signatures might serve as early risk indicators^41^. However, these cross-sectional designs cannot disentangle whether dysbiosis is a cause or a consequence of AD pathology, given that disease progression itself(including reduced dietary intake, altered gut motility, and medication use)can profoundly reshape the microbial ecosystem^15^,^42^.

Longitudinal studies offer stronger, though still limited, inferential power. A prospective cohort study following cognitively normal older adults over several years found that baseline gut microbiota composition, particularly lower abundance of Bifidobacterium and higher abundance of pro-inflammatory Enterobacteriaceae, was associated with an increased hazard of progressing to MCI or AD dementia^43^,^44^. Such findings lend temporal support to a potential causal direction from dysbiosis to neurodegeneration^44^. Nevertheless, these longitudinal observations are often confounded by numerous factors, including diet, polypharmacy (especially proton pump inhibitors, metformin, and antibiotics), comorbid conditions (e.g., diabetes, cardiovascular disease), and socioeconomic status, that independently modulate both the microbiome and cognitive trajectory^42^,^44^. Moreover, the relatively short follow-up durations in existing studies (typically 2-5 years) may not adequately capture the protracted prodromal phase of AD spanning decades^15^,^39^.

### 4.2 Findings from human Fecal Microbiota Transplantation (FMT) studies

The causal role of human gut microbiota has been experimentally tested through FMT into germ-free or microbiota-depleted animal recipients^30^,^31^. In landmark studies, transplantation of fecal microbiota from human AD patients into young adult mice induced recipient animals to exhibit AD-related pathological features, including impaired adult hippocampal neurogenesis, reduced synaptic plasticity markers, and cognitive deficits in behavioral tasks^30^,^45^. These cross-species transfer experiments provide the strongest experimental evidence to date that the human AD-associated microbiota harbors pro-pathogenic properties capable of transmitting disease-relevant phenotypes^31^,^45^.

However, critical caveats apply. First, the translatability of human-to-mouse FMT findings is constrained by substantial species differences in gut microbial composition, metabolic capacity, and immune system architecture^13^,^46^. A microbial community that is dysbiotic in a human host may exert entirely different effects when transplanted into a murine gut with distinct physiological parameters (e.g., body temperature, gut transit time, dietary patterns)^13^,^47^. Second, the quantity of transplanted material required to colonize mice (often administered via repeated oral gavage) far exceeds typical human exposure scenarios^48^. Third, these studies largely use young, healthy recipient animals rather than aged or genetically susceptible hosts, potentially overestimating the effect size of the transplanted microbiota^2^,^46^. Most notably, while human FMT can recapitulate certain AD-related phenotypes in animals, no controlled human FMT trials have yet demonstrated that modifying the microbiota can alter AD progression in patients, representing a fundamental translational gap^15^,^36^.

### 4.3 Metabolomics and inflammatory markers in human cohorts

Human metabolomic studies have partially validated findings from animal models^28^,^29^. Decreased circulating levels of SCFAs, particularly butyrate, have been observed in AD patients compared to controls, and lower SCFA concentrations correlate with higher cerebral Aβ burden measured by amyloid PET or CSF Aβ42 levels^12^,^49^. Similarly, shifts in tryptophan metabolism toward the neurotoxic kynurenine pathway (with elevated kynurenine-to-tryptophan ratio) have been documented in the plasma of AD patients, mirroring preclinical observations^9^,^50^. Furthermore, elevated serum LPS levels and LPS-binding protein (LBP), markers of microbial translocation across a compromised intestinal barrier, have been repeatedly reported in AD cohorts and associate with the severity of cognitive impairment^1^,^11^.

Despite these consistent associations, the effect sizes reported across studies are often modest and overlapping between diagnostic groups, limiting their immediate diagnostic utility^15^,^49^. The specificity of these metabolic signatures to AD versus other neurodegenerative conditions (e.g., Parkinson's disease dementia, frontotemporal dementia) remains largely unexplored^2^,^39^. Additionally, peripheral metabolite levels are influenced by numerous host factors independent of gut microbiota, including renal function (which affects metabolite clearance), dietary intake (particularly of tryptophan and fiber), liver metabolic capacity, and systemic inflammatory status^42^,^51^. Thus, whether observed metabolite changes directly reflect gut microbial metabolism or represent secondary consequences of systemic physiological alterations in AD remains an open question requiring more sophisticated analytical approaches, such as stable isotope tracing in human subjects^3^,^47^.

### 4.4 Major translational barriers and future directions for human research

Several critical barriers currently impede the translation of gut-brain axis findings into clinical practice for AD^15^,^34^:

(1) Causality and Confounding: Establishing causality in humans is inherently difficult due to the reciprocal, dynamic interactions among the microbiota, host metabolism, and disease progression^52^,^53^. Traditional epidemiological approaches cannot fully control for the multitude of confounding variables that shape the gut ecosystem^15^,^42^. Mendelian randomization (MR) using genetic variants as instrumental variables for microbial traits offers a promising strategy to infer causal directions from observational data, but MR studies in AD-microbiome research remain sparse and are limited by the polygenic nature of both traits and the lack of robust genetic instruments for specific microbial taxa^52^-^54^.

(2) Temporal Dynamics and Longitudinal Heterogeneity: The gut microbiota exhibits substantial intra-individual temporal variability influenced by diet, medication, infections, and other environmental exposures^13^,^55^. A single time-point measurement (common in most published AD-microbiome studies) cannot capture this dynamic trajectory^15^,^55^. Consequently, the field lacks understanding of whether microbiota changes precede clinical symptom onset by years or decades (thereby serving as true prodromal biomarkers) or occur relatively late in the disease course (thereby reflecting epiphenomena of advanced neurodegeneration)^43^,^44^.

(3) Lack of Standardization: Methodological heterogeneity across studies, including differences in fecal sampling protocols (spot samples versus timed collections), DNA extraction kits (bead-beating intensity, lysis conditions), 16S rRNA variable region selection (V3-V4 versus V4), sequencing platforms (Illumina versus Nanopore), and bioinformatic pipelines (DADA2 versus QIIME2 versus mothur), complicates cross-study comparisons and meta-analyses^13^,^56^. The absence of universally adopted positive and negative controls (including mock communities and extraction blanks) across most clinical microbiome studies further undermines reproducibility^14^,^56^.

(4) Limited Interventional Evidence: To date, no large-scale, double-blind, randomized controlled trial (RCT) has demonstrated that modulating the gut microbiota (e.g., through probiotics, prebiotics, or FMT) can slow cognitive decline or alter AD biomarkers in human patients with established disease^32^,^33^. Existing small-scale trials have focused on safety, tolerability, and intermediate outcomes (e.g., changes in inflammatory markers, cognitive scale scores), with inconsistent results and high dropout rates^57^,^58^. The optimal formulation (single strain versus multi-strain), dosing regimen, delivery vehicle, and patient stratification criteria remain undefined^59^,^60^. Moreover, the potential for adverse effects, including probiotic-induced bacteremia in vulnerable populations or transmission of pathogenic microbes via FMT, necessitates rigorous safety monitoring^48^,^61^.

Conclusion: Collectively, human clinical evidence strongly supports an association between gut microbiota dysbiosis and AD but has not yet firmly established causality or provided ready-to-use clinical biomarkers^2^,^15^. The field is transitioning from descriptive cross-sectional comparisons toward mechanistic hypothesis testing using longitudinal cohort designs, multi-omics integration, and small-scale interventional proof-of-concept trials^3^,^39^. Overcoming the translational barriers outlined above will require harmonized methodologies, larger and better-phenotyped prospective cohorts with repeated sampling, and rigorous RCTs designed with AD-relevant primary endpoints (e.g., change in CSF p-tau/Aβ42 ratio, amyloid PET standardized uptake value ratio) rather than solely cognitive outcomes^32^,^52^.While the above subsections have summarized the key clinical findings in humans, a more comprehensive and consolidated discussion of the overarching methodological and translational limitations facing the entire field including issues of causality, temporal dynamics, standardization, and the paucity of interventional evidence is presented in Section 9, "Critical Appraisal and Limitations of Current Evidence" (Table 1).

## 5. The Synergistic Role of Imbalance of the Gut Microbiota-Brain Axis and Metabolic Remodeling in the Pathology of AD

### 5.1 The causal association between gut microbiota dysbiosis and AD pathology

Recent research evidence indicates that the dynamic regulatory network formed by the gut microbiota and the central nervous system through the gut-brain axis, and the dysbiosis of its homeostasis has been suggested to be closely associated with, and potentially contributory to, the pathological process of AD, based largely on preclinical models and associative human studies^1^,^2^. Clinical and animal model studies have shown that the gut microbiota of AD patients and transgenic animals (such as APP/PS1, 3xTg, 5xFAD mice) shows significant structural disorders, characterized by a decrease in the abundance of butyrate-producing bacteria (such as the genus Clostridium) and an abnormally high proportion of pro-inflammatory microbiota (such as the genus Bacteroides)^23^,^28^. However, emerging evidence from species- and strain-resolved studies indicates that the functional consequences of Bacteroides colonization in AD cannot be generalized at the genus level, as different Bacteroides species exert markedly distinct, and in some cases opposing, effects on neuropathology^19^,^62^. On the pro-inflammatory and pathogenic side, Bacteroides fragilis is selectively enriched in the gut microbiota of AD patients and has been mechanistically linked to disease pathogenesis^19^,^45^. Colonization of Thy1-C/EBPβ transgenic mice with live (but not heat-killed) B. fragilis induces AD-like pathologies, including microglial activation, upregulation of the C/EBPβ/AEP signaling pathway, and cognitive impairment^19^,^31^. Metabolomic profiling revealed that B. fragilis-derived polyunsaturated fatty acid metabolites, specifically 12-hydroxy-heptadecatrienoic acid (12-HHTrE) and prostaglandin E2 (PGE2), directly activate primary microglia and recapitulate AD pathologies when administered alone^19^,^45^. Furthermore, lipopolysaccharide derived from B. fragilis (BF-LPS) exhibits particularly potent pro-inflammatory and neurotoxic properties compared with LPS from other Gram-negative bacteria, further implicating this species in the propagation of neuroinflammation via the gut-brain axis^19^,^25^.

**Figure 2:**
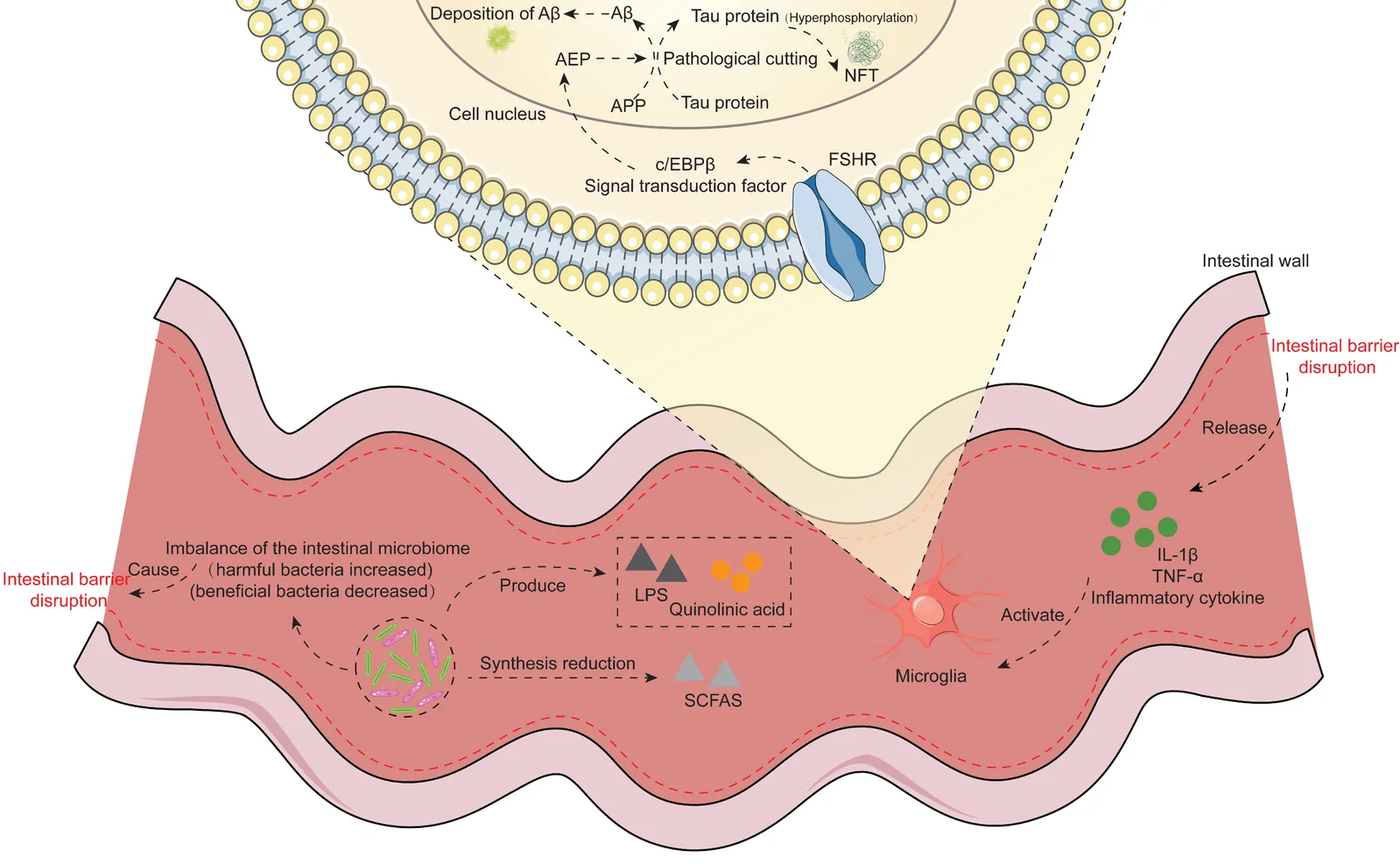
Role of gut microbiota dysregulation in the pathogenesis of Alzheimer's disease: the C/EBPβ-AEP signaling pathway. Dysregulation of the gut microbiota triggers metabolic changes, leading to an increase in LPS, neurotoxin quinolinic acid, etc., while downregulating the synthesis pathway of SCFAs. These changes disrupt the integrity of the intestinal barrier, impairing its function. The damaged intestinal barrier allows pro-inflammatory cytokines (e.g., IL-1β, TNF-α) to enter the bloodstream and cross the blood-brain barrier, activating microglia. Activated microglia promote the pathological cleavage of APP and tau protein via the C/EBPβ-AEP signaling pathway, generating β-amyloid (Aβ) and hyperphosphorylated tau protein. These ultimately form amyloid plaques and NFTs, driving the development of the core pathological features of Alzheimer's disease (AD), and the molecular interactions and regulatory details of the C/EBPβ-AEP pathway are discussed in Section 5.2.

On the other hand, Bacteroides thetaiotaomicron (B. theta), a dominant, mucus-colonizing symbiont in the human gut, exerts region-specific and complex effects on AD-related traits^62^. Mono-colonization of germ-free mice with B. theta reduces the secretion of neuroprotective sAPPα in the hippocampus while increasing presynaptic bouton numbers in the same region, but has opposite or neutral effects in the prefrontal cortex and cerebellum, suggesting brain region-dependent modulation of AD-related outcomes^62^. Intriguingly, individuals with elevated cerebral Aβ burden exhibit a notable association between B. thetaiotaomicron and butyrate production, indicating that the metabolic output, and hence the functional consequence, of this species may depend on the broader microbial community context and host metabolic state^17^,^62^. Even within the same Bacteroides species, strain-level genomic variations (e.g., differences in polysaccharide utilization loci, capsular biosynthesis gene clusters, and metabolite production profiles) can profoundly alter host-microbe interactions and disease outcomes, underscoring the necessity of moving beyond genus-level taxonomic assignments in gut-brain axis research^13^,^14^. This microbiota imbalance damages the integrity of the intestinal barrier, induces the systemic translocation of LPS and pro-inflammatory cytokines (IL-6, TNF-α). This systemic inflammatory burden further compromises blood-brain barrier integrity and exacerbates neuroinflammation via the LPS-TLR4/NF-κB axis (see Section 5.2 for the detailed microglial signaling cascade), contributing to Aβ deposition^1^,^25^,^26^. Evidence from animal models provides experimental support for this potential association. For example, after transplanting the fecal microbiota of AD patients into germ-free 3xTg mice, the Aβ plaque load in the brains of the recipient animals significantly increases, accompanied by impaired adult hippocampal neurogenesis and deficits in spatial memory, while the transplantation of the microbiota from healthy donors could ameliorate some pathological phenotypes in these models^30^,^31^. It is crucial to note, however, that these causal relationships established in animal models cannot be directly extrapolated to humans without further longitudinal and interventional studies in clinical settings^2^,^15^. Mechanistic investigations in AD mouse models have demonstrated that gut microbiota dysbiosis can trigger the C/EBPβ-AEP signaling axis-a critical node that promotes amyloid precursor protein (APP) pathological cleavage and tau hyperphosphorylation. Given its central role, the upstream regulatory network and downstream effectors of this pathway are comprehensively dissected in Section 5.2^20^,^31^,^63^. However, direct evidence of this mechanism in the human AD brain remains to be established^15^,^39^. while this pathway represents a compelling mechanistic link, its precise role and activity in the human AD brain remain to be fully elucidated (see detailed mechanistic analysis in Section “Molecular Mechanisms of Metabolic Reprogramming and the Regulation of Neurodegeneration”). In addition, the systemic imbalance of microbiota metabolites (such as the decrease of SCFAs and the accumulation of LPS), which are hypothesized to drive neurodegeneration through multiple interrelated pathways (detailed in Section 5.2) can accelerate neuronal degeneration by inducing oxidative stress, mitochondrial dysfunction and immunoregulatory disorders^4^,^57^.These findings provide a conceptual framework suggesting that gut microbiota dysbiosis may play a role in the pathological evolution of AD through a local-central multi-level regulatory network^1^,^3^. However, most of the mechanistic insights are derived from preclinical models, and translation to human pathology requires further investigation^2^,^15^ (Fig. 2).

### 5.2 The molecular mechanisms of metabolic reprogramming and the regulation of neurodegeneration

The interactive remodeling (metabolic reprogramming) between the gut microbiota and the host metabolic network has been proposed as a potential key contributor to the pathological progression of AD, based on accumulating preclinical and correlational clinical evidence^4^,^64^,^65^. At the level of SCFAs metabolism, the reduced synthesis of SCFAs such as butyrate not only impairs the function of the intestinal epithelial barrier, but also regulates the phenotypic polarization of microglia and neuroinflammatory responses by inhibiting the activity of histone deacetylases (HDACs)^22^,^66^. In addition, SCFAs regulate the sensitivity of the insulin signaling pathway in the brain and neuronal survival by activating GPR41/43 receptors, and the imbalance of their metabolism is closely related to the impairment of synaptic plasticity associated with AD^7^,^21^. Notably, an emerging paradigm challenges the simplistic "good vs. bad" dichotomy of microbial metabolites: concentration-dependent biphasic (hormetic) effects. For butyrate, physiological concentrations (≤2 mM) exert neuroprotection via HDAC inhibition, whereas supraphysiological levels (≥10 mM) may induce mitochondrial dysfunction and oxidative stress in specific neuronal subtypes-a finding that complicates oral butyrate supplementation strategies. Similarly, kynurenic acid is neuroprotective at low doses but exacerbates excitotoxicity upon quinolinic acid accumulation. This hormetic property implies that future postbiotic interventions must precisely titrate metabolite concentrations within a therapeutic window, rather than pursuing unidirectional "replenishment." the shift of tryptophan towards the kynurenine pathway, which can be influenced by the gut microbiota in animal models, has been associated with the accumulation of the neurotoxic metabolite quinolinic acid^9^,^50^. Preclinical studies suggest that this metabolite may contribute to neuronal damage by activating oxidative stress and promoting Aβ aggregation, although direct human evidence is still limited^9^,^50^. At the same time, the reduced synthesis of 5-hydroxytryptamine (5-HT) may further impair the efficiency of synaptic transmission and aggravate cognitive impairment^8^,^50^,^67^. Lipid metabolic remodeling also plays a key role in the pathology of AD. gut microbiota dysbiosis affects the homeostasis of cholesterol in the brain and the function of apolipoprotein E (ApoE) by regulating the metabolism of bile acids, thus promoting the formation of Aβ oligomers and the spread of tau pathology^64^,^68^. In addition, the abnormal sphingolipid metabolic pathway is closely related to microbiota-mediated mitochondrial dysfunction, and its metabolites directly participate in the neurodegenerative process by activating the neuronal apoptosis signaling pathway^64^,^69^. It is worth noting that microbiota-derived metabolites (such as LPS, hydrogen sulfide) can induce the polarization of microglia towards the pro-inflammatory M1 phenotype by activating the TLR4/NF-κB signaling axis, and continuously release inflammatory factors such as IL-1β and TNF-α, ultimately forming a chronic neuroinflammatory microenvironment^1^,^25^,^45^,^70^. Despite the robustness of these mechanistic pathways in preclinical models, direct evidence linking specific microbial metabolites to these effects in the human AD brain is still lacking^2^,^15^. Most current data are derived from correlational analyses in human cohorts or interventional studies in animals, and causality or the relative contribution of each pathway remains to be determined in a clinical context^3^,^39^.

**Figure 3:**
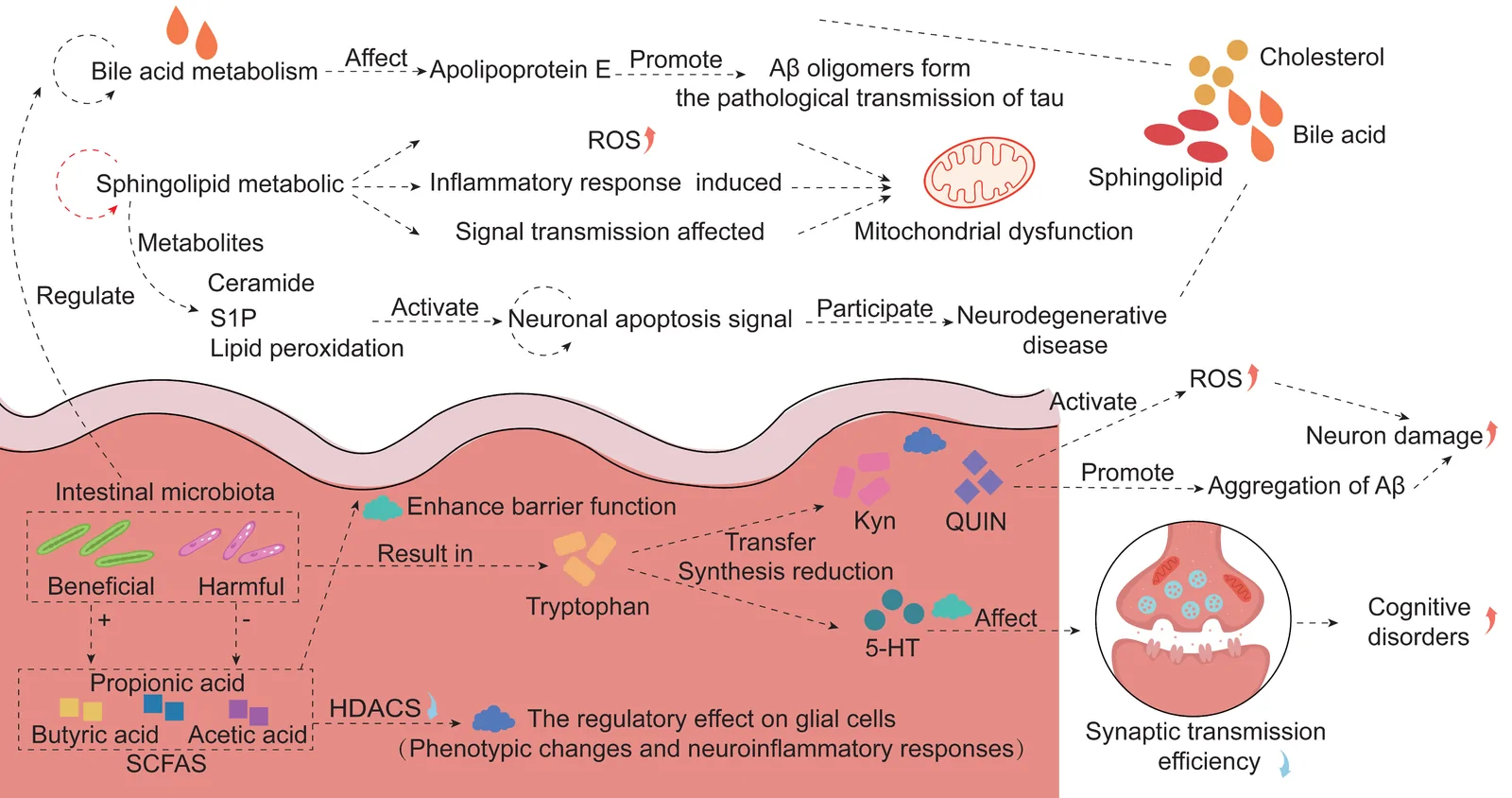
Mechanisms underlying the association between gut microbiota metabolic reprogramming and neurodegenerative diseases. Changes in the composition of the gut microbiota affect short-chain fatty acid (SCFA) metabolism, tryptophan metabolic pathways, and lipid metabolism. Reduced SCFA synthesis impairs intestinal epithelial barrier function, regulates microglial phenotypic polarization and neuroinflammatory responses by inhibiting histone deacetylase (HDAC) activity, and influences the sensitivity of insulin signaling pathways in the brain and neuronal survival. In the tryptophan metabolic pathway, a shift toward the kynurenine pathway leads to accumulation of the neurotoxic metabolite quinolinic acid (QUIN), which activates oxidative stress and promotes Aβ aggregation, exacerbating neuronal damage; decreased serotonin (5-HT) synthesis impairs synaptic transmission efficiency and aggravates cognitive impairment. In terms of lipid metabolism remodeling, gut microbiota dysregulation affects cholesterol homeostasis and ApoE function by regulating bile acid metabolism, promoting Aβ oligomer formation and tau pathology propagation; abnormal sphingolipid metabolic pathways are associated with mitochondrial dysfunction, and their metabolites directly participate in neurodegenerative processes by activating neuronal apoptotic signaling pathways. These metabolic changes collectively drive the progression of neurodegenerative diseases.

Recent systems biology studies have revealed that the four canonical pathways (SCFAs-HDAC, tryptophan-kynurenine, C/EBPβ-AEP, and LPS-NF-κB) do not function independently but form a highly interconnected regulatory network^31^,^45^. Mechanistically, LPS-induced NF-κB activation directly upregulates C/EBPβ expression, creating a feed-forward loop that amplifies AEP-mediated APP and tau cleavage^19^,^31^. Concurrently, the shift of tryptophan metabolism toward the kynurenine pathway generates quinolinic acid, which further sensitizes microglial NF-κB signaling, while SCFAs deficiency relieves HDAC-mediated repression of pro-inflammatory gene transcription, collectively establishing a chronic neuroinflammatory microenvironment^22^,^50^. Notably, a hierarchical temporal relationship among these pathways has been identified in longitudinal animal studies: gut dysbiosis-induced LPS elevation precedes NF-κB activation (occurring at 2-4 months in 5xFAD mice), followed by C/EBPβ-AEP upregulation (4-6 months), while SCFAs decline and kynurenine pathway shift manifest as early as 1-2 months, suggesting they may serve as initiating events^23^,^31^. This temporal hierarchy provides a rationale for stage-specific intervention strategies targeting distinct pathways at different disease phases^20^,^33^. The disorders of these multi-dimensional metabolic pathways not only highlight the pivotal position of the gut microbiota in metabolic regulation in AD, but also provide potential molecular targets for targeted intervention^3^,^32^. Notably, recent studies have moved beyond a binary “good vs. bad” view of microbiota-derived metabolites, revealing their concentration-dependent and tissue-specific effects^9^,^21^. Taking butyrate as an example, while physiological concentrations exert neuroprotection via HDAC inhibition, the paradox of severe butyrate deficiency in the gut of AD patients alongside its abnormal accumulation in certain brain regions suggests a potential hormetic (bell-shaped) effect^21^,^49^,^71^. Similarly, the tryptophan metabolite kynurenic acid is neuroprotective at low concentrations (via antioxidant properties) but can exacerbate excitotoxicity at high levels, synergizing with quinolinic acid^9^,^50^. This functional duality indicates that simple “replenishment or depletion” strategies may be insufficient; future interventions require precise control of metabolite concentrations within a therapeutic window^33^,^59^. This insight imposes higher demands on postbiotic design - maintaining a therapeutically effective concentration range in target tissues (e.g., local gut or central nervous system) while avoiding inverse effects caused by oversupplementation^21^,^33^ (Fig. 3).

Notably, recent studies have revealed a concentration-dependent bifacial effect of gut microbiota-derived metabolites on AD pathology. For short-chain fatty acids (SCFAs), physiological concentrations of butyrate (≤ 2 mM) exert neuroprotection via HDAC inhibition, whereas high concentrations (≥ 10 mM) can induce neurotoxicity characterized by loss of mitochondrial membrane potential and excessive ROS production. Similarly, the tryptophan metabolite kynurenic acid (KYNA) is neuroprotective as an NMDA receptor antagonist at low levels, but a shift toward the QUIN pathway results in excitotoxicity. This “concentration-effect” biphasicity challenges simple metabolite-boosting strategies: clinical benefit may require spatiotemporally precise regulation of metabolite levels in specific brain regions and cell types rather than global elevation.

## 6. Integrated Analysis of Multi-omics

**Figure 4:**
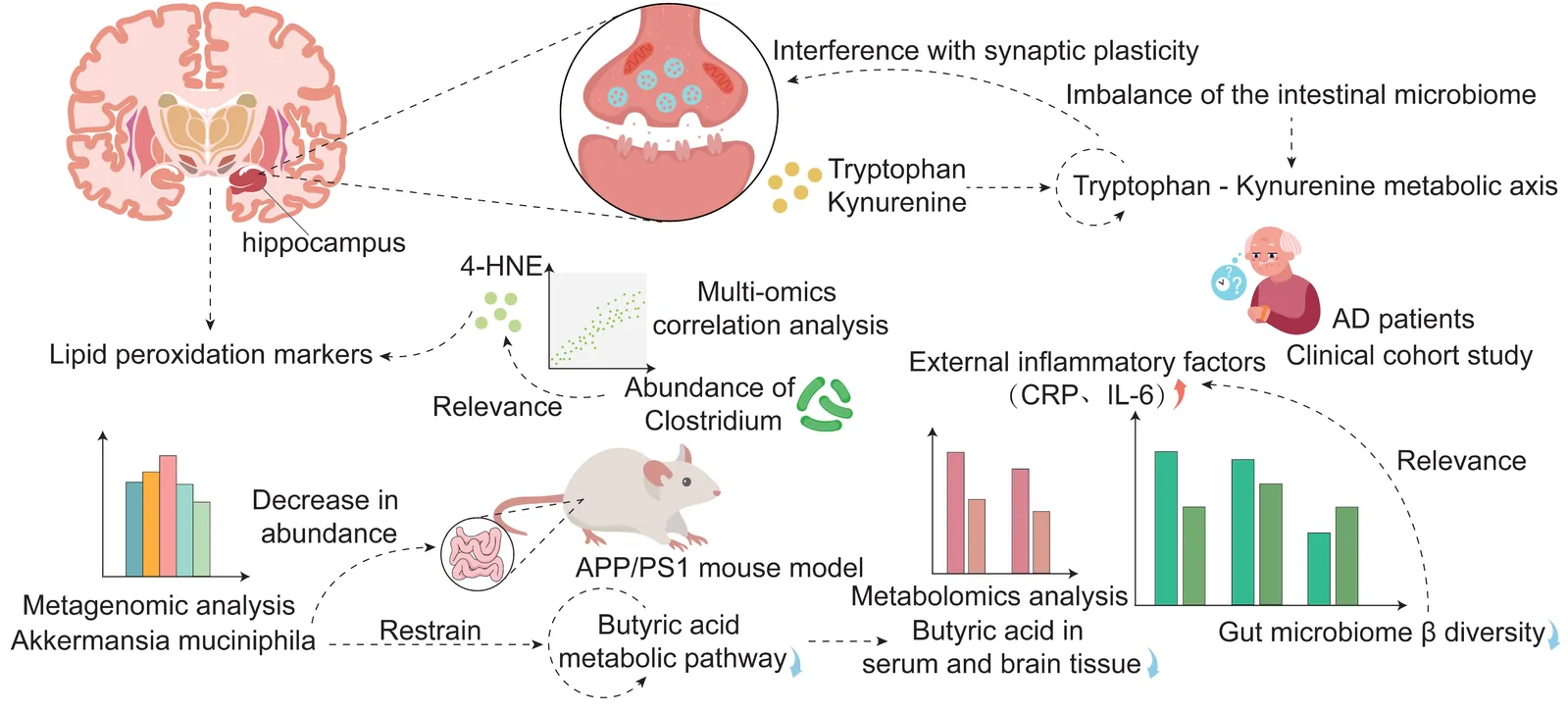
Regulation network of the gut-brain axis in AD revealed by multi-omics technology integration. Integrated analysis using multi-omics technologies shows that the reduced abundance of the mucus-degrading bacterium Akkermansia muciniphila in the APP/PS1 mouse model is associated with the inhibition of butyrate metabolic pathways. Metabolomics data confirm a decrease in butyrate levels in serum and brain tissues. Spatial metabolomics and transcriptomics studies indicate that gut microbiota dysregulation disrupts synaptic plasticity in the hippocampus via the tryptophan-kynurenine metabolic axis, providing mechanistic evidence for the regulation of cognitive functions by the microbiome. Cross-omics correlation analysis reveals a significant association between increased abundance of Clostridium and elevated levels of the lipid peroxidation marker 4-HNE in the brain, suggesting that oxidative stress serves as a core hub linking gut microbiota dysregulation and neurometabolic disorders. Clinical cohort studies show that reduced gut microbiota β-diversity is associated with increased levels of peripheral inflammatory factors (e.g., CRP, IL-6), supporting the critical role of the "microbiome-immunity-brain axis" in AD progression. These findings uncover the complex connections between the gut microbiota and AD pathological processes, highlighting the importance of multi-omics technologies in deciphering the regulatory network of the gut-brain axis.

The integrated application of multi-omics technologies (metagenomics, metabolomics, transcriptomics and proteomics) provides a new paradigm for systematically analyzing the regulatory network of the gut-brain axis in AD^28^,^29^. In the APP/PS1 mouse model, metagenomic analysis shows a significant association between the decrease in the abundance of the mucin-degrading bacterium Akkermansia muciniphila and the inhibition of the butyrate metabolic pathway, metabolomic data further confirms the decrease in the levels of butyrate in serum and brain tissues (consistent with the SCFAs-related mechanisms discussed earlier)^28^,^29^. Spatial metabolomics and transcriptomics have provided high-resolution validation of the tryptophan-kynurenine pathway dysregulation in the hippocampal region, providing molecular evidence with spatial resolution for the regulation of cognitive functions by the microbiota^28^,^50^. Cross-omics correlation analysis has identified significant associations that suggest potential synergistic influences on AD pathology^28^,^64^. However, these statistical associations from correlational analyses do not imply causation^13^,^15^. For example, in the 5xFAD mouse model, the increase in the abundance of the genus Clostridium is significantly correlated with the elevated level of the lipid peroxidation marker 4-HNE in the brain, suggesting that oxidative stress is the core hub connecting microbiota dysbiosis and neuro-metabolic disorders^23^,^64^. In clinical cohort studies, the integrated analysis of fecal metagenomics and plasma metabolomics shows a strong correlation between the decrease in the β-diversity of the gut microbiota and the increase in the levels of peripheral inflammatory factors (CRP, IL-6), supporting the crucial role of the "microbiota-immune-brain axis" in the progression of AD^10^,^11^. Although multi-omics association analyses have constructed large-scale networks linking microbiota, metabolites, and host phenotypes, a critical bottleneck remains: distinguishing causality from correlation^15^,^52^. Most current strategies are limited to descriptive associations, lacking a systematic functional validation loop^3^,^39^. To overcome this bottleneck, a “multi-omics guided mechanistic validation” framework has recently been proposed^47^,^51^. This framework involves three steps: (1) screening for metabolites strongly associated with core AD pathologies (e.g., TMAO, imidazole propionate) in cohort studies; (2) colonizing germ-free or antibiotic-treated AD model mice with single bacterial strains or administering specific metabolites to validate causal effects on Aβ deposition or tau pathology; and (3) constructing metabolite-pathway-knockout bacterial strains using CRISPR interference to reversely verify necessity of the metabolic pathway^51^,^72^. For example, using this strategy, recent work demonstrated that microbiota-derived imidazole propionate directly binds and activates p38 MAPK, promoting tau phosphorylation at Ser202/Thr205^20^,^51^. This “correlation-causality-mechanism” closed-loop paradigm represents the future gold standard for gut-brain axis research^18^,^29^. Although significant progress has been made in multi-omics technologies, current research still faces challenges such as sample heterogeneity, complexity of data integration and limitations of causal inference^28^,^73^. In the future, it is necessary to combine longitudinal cohort studies, germ-free animal models and advanced bioinformatics approaches to dynamically analyze the spatiotemporal specific regulatory mechanisms of specific strains or metabolites (such as butyrate, quinolinic acid) on AD pathology^23^,^74^. Machine learning and artificial intelligence techniques have shown potential in integrating multi-omics data for AD biomarker discovery and patient stratification, but such applications remain in exploratory research stages^33^,^35^. Their clinical implementation will require overcoming major challenges including methodological heterogeneity, inter-individual microbiome variation, data governance issues, and algorithmic bias^35^,^74^. Nevertheless, continued refinement of these computational approaches may ultimately contribute to the development of more precise diagnosis and treatment strategies in the long term^3^,^38^.The integration of multi-omics not only deepens the understanding of the complex network of the gut-brain axis, but also provides a new direction for the discovery of early biomarkers and targeted treatment of AD^27^,^32^ (Fig. 4).

Traditional multi-omics association analyses often struggle to distinguish correlation from causation^15^,^52^. Recently, machine learning-based causal inference frameworks (e.g., causal Bayesian networks, structural equation modeling combined with MR) have been successfully applied to AD microbiome research^73^,^74^. Using two-sample MR, demonstrated a causal relationship between relative abundances of Eubacterium and Ruminococcus and AD risk (OR = 1.32, 95% CI: 1.12-1.56)^53^,^54^,^75^. Moreover, deep learning-based counterfactual models can simulate how perturbations of specific microbial metabolic pathways (e.g., butyrate synthesis) affect Aβ deposition dynamics, identifying actionable targets^73^,^74^. Future integration of longitudinal multi-omics with time-series causal models will help pinpoint core drivers along the cascade of “dysbiosis → metabolic disruption → neuroinflammation → cognitive decline.”^44^,^52^

## 7. Synergistic Regulatory Network from a Multidisciplinary Integration Perspective

The pathological evolution of AD involves the interaction of multiple systems and functional disorders at multiple levels^2^,^3^. Its complexity requires a shift in the research paradigm from a single discipline to interdisciplinary integration^27^,^38^. Through the cross-integration of medicine, biology, microbiology, and computational science, researchers have gradually revealed the systematic association between the clinical heterogeneity of AD, the imbalance of microbiota metabolism, and the dynamic changes of molecular interaction networks, providing an innovative framework for in-depth analysis of the disease regulatory mechanisms^3^,^39^.

### 7.1 Medical perspective

The heterogeneity of the clinical manifestations of AD is significantly related to the diversity of its pathophysiological mechanisms, and the systematic regulation of gut microbiota metabolites may serve as a key hub connecting host metabolic disorders and the neurodegenerative process^1^,^64^. Cohort studies have shown that the α-diversity of the gut microbiota in AD patients with insulin resistance is significantly reduced, and the interaction network between the microbiota composition and host metabolic markers (such as adiponectin and oxidized lipids) is reconstructed, suggesting that the imbalance of the microbiota-metabolic axis plays a crucial role in the pathology of AD^44^,^64^. Longitudinal studies have confirmed that the dynamic changes of inflammatory markers such as IL-6 and CRP in the circulatory system are significantly related to the clinical stages of AD, and SCFAs derived from the gut microbiota can affect the spatiotemporal progression of neuroinflammation via multifaceted mechanisms, including the modulation of blood-brain barrier integrity and microglial metabolic reprogramming (the detailed molecular pathways are elaborated in Section 5.2)^1^,^22^. It is worth noting that multicenter studies based on the Asian population have revealed that the abundance changes of gene clusters such as PLEC and C1Q in the gut microbiota of AD patients are significantly correlated with the Aβ42/tau ratio in cerebrospinal fluid, suggesting that microbiota metabolism may regulate the pathological phenotypes of the central nervous system through the molecular interaction network^10^,^17^.

### 7.2 Biological perspective

The research on the molecular mechanisms of AD has shifted from single-target analysis to dynamic modeling of multi-level networks^4^,^64^. The molecular interaction network constructed by integrating transcriptomic, phosphoproteomic, and metabolomic data shows that gene modules related to synaptic plasticity and mitochondrial oxidative phosphorylation exhibit significant functional disorders in the early stage of the disease^4^,^64^. Protein interaction network analysis based on the STRING database has found that core nodes such as APOE and TREM2 form a synergistic regulatory module by regulating lipid metabolic reprogramming and the phagocytic function of myeloid cells, and its dynamic imbalance is closely related to the clinical trajectory of the conversion from MCI to AD dementia^4^,^16^,^76^. In addition, dynamic metabolic flux analysis technology (such as the COMETS platform) can simulate the metabolic complementary network of the microbial community^77^. Combined with the temporal change data of host metabolic markers, it provides a new tool for the quantitative analysis of the "microbiota-immune-metabolism" cascade reaction in AD^47^,^77^,^78^.

### 7.3 Integration of microbiology and computational biology

The integration of microbiomics and computational biology provides a valuable analytical framework for AD research^28^,^73^. Systematic analysis combining metagenomics with metabolic network modeling (such as the iNAP 2.0 platform) has successfully mapped the gut microbial tryptophan metabolic network. As a corollary of the kynurenine pathway shift (described in Section 5.2), this modeling approach links reduced 5-HT synthesis to hippocampal synaptic dysfunction^28^,^50^.

Looking to future directions, advanced computational methods hold potential for deeper integration of multi-omics data. As an extension of the analytical strategies outlined in Section 6, exploratory studies have begun applying machine learning to gut microbiota data for dementia diagnosis; a recent scoping review identified several predictive models, including random forests and neural networks, for detecting AD-associated microbial alterations^73^,^74^. However, the authors of that review concluded that current literature lacks a comprehensive framework for clinical translation, and that elucidating gut-brain interactions for diagnostic purposes remains an ongoing challenge^39^,^73^. Preliminary work on multimodal AI models that integrate blood metabolites, brain imaging, microbiome profiles, and clinical data has achieved promising accuracy (approximately 75%) in predicting APOE4 genotype in research settings, but these models require validation in larger, diverse cohorts before clinical application^35^,^74^.

Similarly, in the more distant future, synthetic biology tools such as CRISPR-based gene editing may accelerate the identification of potential intervention targets^72^,^79^. However, it is important to note that CRISPR-engineered probiotics have only been tested in fruit fly models to date, and no human clinical trials have been initiated for AD^72^,^79^. Significant barriers (including safety concerns, regulatory approval processes, strain stability, colonization efficiency, and public acceptance) must be addressed before such approaches can be considered for clinical translation^34^,^48^.

Although interdisciplinary research has revealed the synergistic regulatory rules of the "clinical phenotype-microbiota metabolism-molecular network" in AD, core issues such as data heterogeneity, model complexity, and limitations of causal inference still need to be addressed^15^,^73^. Future research needs to integrate single-cell spatiotemporal omics and dynamic data of microbial communities to develop cross-scale models that can simultaneously depict the interaction between the host and microbiota and the evolution of molecular networks^[80.81]^. In addition, personalized intervention strategies based on network pharmacology (such as targeted regulation of microbiota metabolism combined with immunomodulation) need to verify their translational potential through prospective clinical trials^32^,^33^. This research paradigm that integrates medicine, systems biology, and artificial intelligence will open up new paths for the precise typing and targeted treatment of AD^27^,^38^.

## 8. Precision Intervention Strategies Driven by Multidisciplinary Intersection

### 8.1 Optimization bottlenecks and systematic challenges of existing intervention strategies

The optimization of current multidisciplinary intervention strategies focuses on the refined reconstruction of the behavior intervention framework and the dynamic adaptation improvement of the collaboration mechanism^32^,^33^. However, its transformation efficiency is still limited by the separation of theories and practices among disciplines^15^,^34^. Current intervention strategies targeting the gut microbiota in AD face several systematic challenges^32^,^59^. First, technical integration barriers remain prominent: while multi-omics analyses have identified characteristic alterations in AD gut microbiota, such as reduced butyrate-producing genera (Faecalibacterium, Roseburia) and increased pro-inflammatory taxa (Escherichia/Shigella), translating these signatures into clinical practice is hindered by a lack of standardized protocols for sample collection, sequencing, and data analysis across different cohorts^13^,^56^. Second, dynamic response defects are evident: most existing probiotic or prebiotic intervention studies lack longitudinal monitoring of both microbiota composition and AD core biomarkers (e.g., Aβ42/tau ratio in cerebrospinal fluid or plasma phosphorylated tau), making it difficult to assess the temporal relationship between microbiota remodeling and cognitive outcomes^32^,^57^. Third, limitations of the evaluation system persist: current efficacy indicators often fail to integrate multi-dimensional data, such as simultaneously quantifying changes in fecal short-chain fatty acid (SCFA) levels, neuroinflammatory markers (e.g., IL-6, TNF-α), and cognitive assessment scores^33^,^58^. Addressing these bottlenecks requires the development of unified technical standards, dynamic systems modeling of host-microbe interactions, and composite evaluation systems that incorporate both microbial and neurological endpoints^34^,^59^. These challenges highlight the deep-seated contradictions in multidisciplinary collaboration, suggesting that in the future, through technological standardization, dynamic system modeling, and innovation of the comprehensive evaluation system, the intervention strategies should be promoted towards precision and scalability^32^,^38^,^82^. Beyond these, two underappreciated translational barriers warrant explicit attention. First, safety surveillance remains severely underdeveloped: FMT has been linked to fatal transmission of ESBL-producing E. coli in other indications, yet AD-specific FMT studies rarely implement comprehensive metagenomic screening for antimicrobial resistance genes or long-term inflammatory biomarker monitoring. Second, methodological standardization across multi-omics studies is alarmingly poor inter-laboratory reproducibility for gut microbiota composition analysis using identical fecal samples is only 47-62%, owing to disparate DNA extraction kits, 16S variable regions, and bioinformatic pipelines. Adopting unified IHMS or MIxS-BE standards is imperative for meaningful cross-study validation.

Current microbiota-based interventions (probiotics, prebiotics, postbiotics, FMT) have shown preclinical promise, but their clinical translation faces two core challenges: efficacy heterogeneity and poorly defined dose-response relationships^32^,^59^. Efficacy heterogeneity arises primarily from substantial inter-individual variation in baseline microbiota composition - colonization efficiency of the same probiotic strain can differ by more than two orders of magnitude among subjects, and metabolic output is constrained by host-resident “colonization resistance”^13^,^60^. Nonlinear dose-response relationships further complicate intervention design. For example, in 3xTg-AD mice, low-dose butyrate (50 mg/kg/d) improved synaptic plasticity, whereas a high dose (200 mg/kg/d) paradoxically induced endoplasmic reticulum stress and apoptosis in the hippocampus^21^,^23^. Similarly, FMT exhibits a U-shaped dose-effect curve, indicating a narrow therapeutic window^48^. These findings emphasize that future interventional studies must incorporate personalized pharmacokinetic/pharmacodynamic modeling, using longitudinal dynamic monitoring of microbiota metabolites to establish an individual-specific “efficacy-toxicity” dose window, rather than adopting a universal fixed-dose regimen^33^,^35^.

### 8.2 Interdisciplinary collaborative design and transformation bottlenecks of emerging therapies

**Figure 5:**
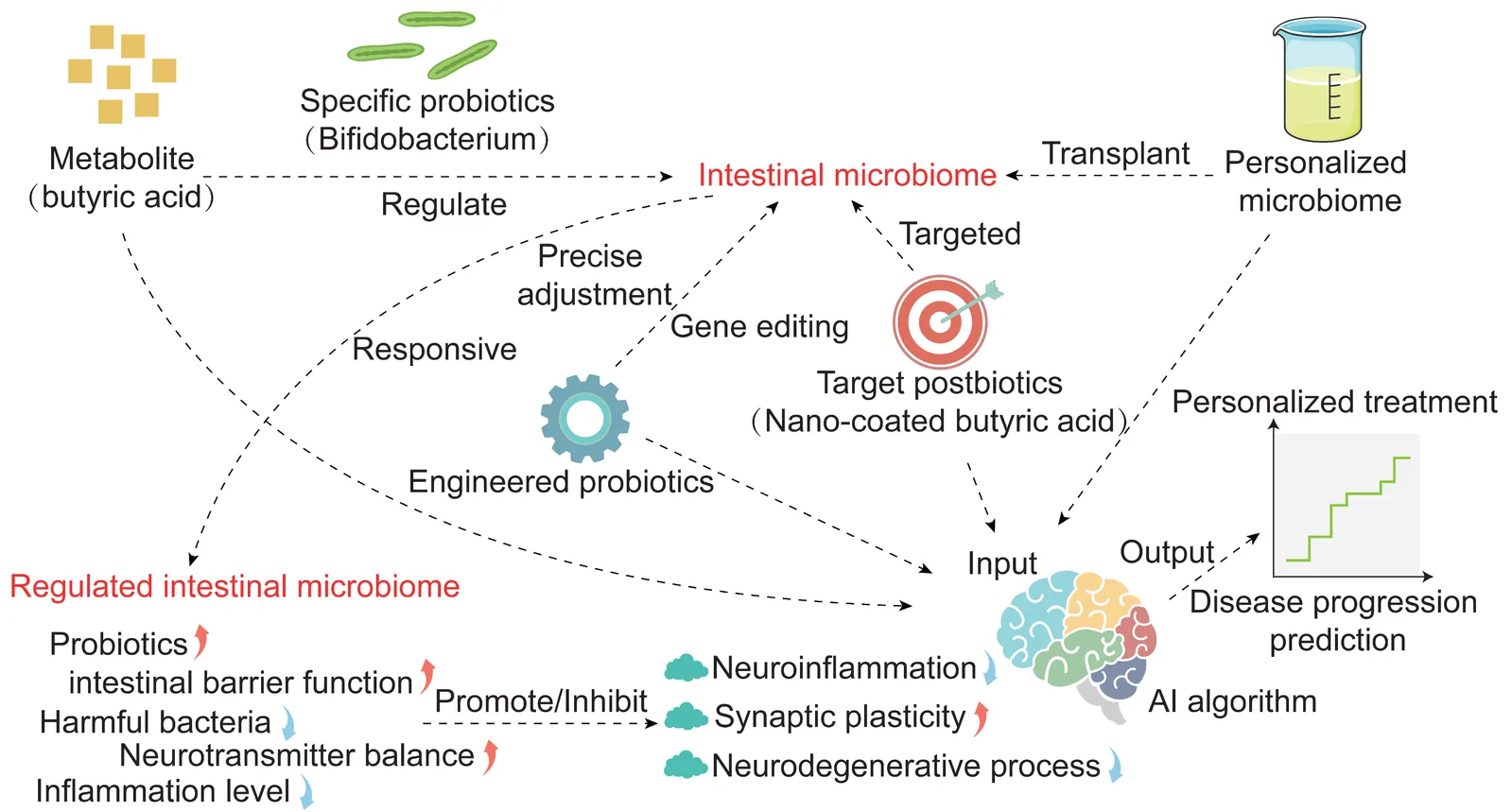
Mechanisms of intervention strategies targeting the gut-brain axis in Alzheimer's disease (AD): current evidence and future directions. This figure illustrates the conceptual framework of gut-brain axis-targeted intervention strategies for Alzheimer's disease. Currently available or clinically investigated approaches include specific probiotics, postbiotics (e.g., butyrate, SCFAs), synbiotics, and dietary interventions that can modulate gut microbiota composition, enhance intestinal barrier function, reduce systemic inflammation, and improve neurotransmitter balance, thereby potentially alleviating neuroinflammation and enhancing synaptic plasticity in preclinical and early clinical studies. Emerging approaches that remain under preclinical investigation include engineered probiotics developed through synthetic biology, which aim to achieve more targeted regulation of the gut microbiota; however, to date, no gene-edited probiotics have entered clinical trials for AD, with current evidence limited to proof-of-concept studies in animal and invertebrate models. Similarly, FMT has shown preliminary cognitive benefits in small-scale human studies, but rigorous randomized controlled trials are needed to establish its efficacy and safety for AD management. Artificial intelligence and machine learning are being explored as tools for multi-omics data integration and biomarker discovery in research settings, but their application in personalized treatment planning and efficacy prediction remains at an exploratory, hypothesis-generating stage and requires extensive prospective validation. Collectively, these intervention strategies represent a spectrum of approaches ranging from clinically testable modalities to longer-term technological frontiers.

The innovative paradigm of emerging treatment technologies is shifting from single-discipline breakthroughs to interdisciplinary collaborative design, and its R&D efficiency depends on the deep integration of cross-domain knowledge and the organic connection of the technological innovation chain^3^,^34^. The development of emerging microbiome-based therapies for AD necessitates a tightly integrated interdisciplinary approach, yet significant translational bottlenecks persist^32^,^34^. Engineered probiotics represent a promising strategy, where strains such as Escherichia coli Nissle 1917 have been genetically modified to express human anti-inflammatory cytokines (e.g., IL-10) or to degrade Aβ peptides locally in the gut^72^,^79^. However, less than 30% of such preclinical studies include comprehensive assessments of both gut barrier integrity and central nervous system (CNS) pathology, highlighting a gap between microbial engineering and neurobiology^15^,^34^. Similarly, targeted postbiotic delivery systems, such as nanoencapsulated butyrate, have demonstrated the ability to inhibit histone deacetylase (HDAC) and reduce neuroinflammation in APP/PS1 mice^21^,^33^. Nevertheless, challenges remain in achieving site-specific release in the colon, crossing the blood-brain barrier (BBB) at therapeutic concentrations, and scaling up production under good manufacturing practice (GMP) standards^48^,^59^. Personalized FMT protocols have shown preliminary success in improving cognitive scores in small-scale AD patient cohorts, but the long-term safety, donor selection criteria, and standardization of administration routes are yet to be established in multicenter, double-blind, placebo-controlled trials^36^,^48^. The key paths to breaking these bottlenecks include: (1) establishing interdisciplinary platforms that integrate synthetic biology, neuroimmunology, and clinical trial design from the outset^34^,^48^; (2) developing artificial intelligence (AI)-driven models to predict individual patient responses based on baseline microbiota composition and metabolic profiles^35^,^74^; and (3) creating shared databases of adverse events and efficacy outcomes to accelerate regulatory approval and clinical adoption^34^,^48^. In the context of AD, the integration of neuroimaging and gut microbiota data represents a paradigm-shifting interdisciplinary approach^12^,^27^. Recent studies have demonstrated that gut microbiota composition correlates with brain glucose metabolism measured by 18F*-*FDG-PET in AD patients, particularly in the hippocampal and posterior cingulate regions^12^,^17^. Furthermore, resting-state fMRI analyses have revealed that decreased abundance of butyrate-producing bacteria (e.g., Faecalibacterium prausnitzii) is significantly associated with reduced functional connectivity within the default mode network (DMN), a key neural signature of early AD^12^,^83^. These imaging-microbiome associations provide non-invasive biomarkers for early diagnosis and suggest that microbiota-derived metabolites may modulate neural network integrity through the gut-brain axis^12^,^27^. Machine learning models integrating multimodal neuroimaging and metagenomic data have achieved improved accuracy in distinguishing AD from MCI, with area under the curve (AUC) values reaching 0.89-0.92^35^,^74^. The current main bottlenecks lie in the lack of interdisciplinary ethical review standards (such as data privacy protection for neuroimmunomodulatory therapies) and insufficient technological standardization (such as the quality control system for organoid culture), and policy guidance and platform construction are needed to accelerate the implementation of solutions^34^,^56^ (Fig. 5).

### 8.3 Frontier directions and collaborative paths of interdisciplinary technology integration

The integration of frontier technologies is being explored in several directions, though most remain in early research phases^73^,^74^. Engineering-driven approaches such as intelligent biomaterials and microfluidic chips are under preclinical investigation for controlled drug delivery^80^,^81^. Computational approaches for multi-omics data integration (including machine learning models that combine clinical, dietary, and microbiome data) have been evaluated for AD risk prediction in research settings^35^,^74^. For example, a recent study using multi-modal machine learning demonstrated that medical history and dietary patterns achieved predictive performance (AUC = 0.871 and 0.874, respectively) in distinguishing AD cases from controls; however, microbiome data in that study were analyzed at an exploratory level only, and the authors emphasized the need for validation in larger cohorts^35^,^74^.

Looking forward, the integration of multi-omics data with advanced computational methods holds conceptual promise for enhancing biomarker discovery and patient stratification, provided that major challenges (including data harmonization, analytical standardization, and prospective clinical validation) are systematically addressed^15^,^56^.

The advancement of precision interventions targeting the gut-brain axis in AD relies on the synergistic integration of frontier technologies within three core directions^32^,^33^. First, AI-driven multi-omics integration: Building upon the correlation analyses in Section 6, machine learning algorithms are now being applied to move from descriptive associations towards predictive modeling. For example, random forest classifiers have been used to identify discriminatory microbial panels, though overfitting and lack of external validation remain major limitations^35^,^73^,^74^. Second, next-generation metabolic modeling: Platforms such as COMETS (Computation of Microbial Ecosystems in Time and Space) enable the simulation of cross-feeding networks and metabolite fluxes within the gut microbial community^77^. Applied to AD, these models can predict how dietary interventions or probiotic administration alter SCFA and tryptophan metabolite production, and subsequently impact CNS inflammation^47^,^77^. Future work must integrate these models with host metabolic data from cerebrospinal fluid (CSF) to capture gut-CNS crosstalk accurately^47^,^84^. Third, advanced in vitro and in vivo models: Human gut-on-a-chip systems combined with induced pluripotent stem cell (iPSC)-derived microglia offer a platform to study the effects of specific microbial metabolites (e.g., LPS, butyrate, quinolinic acid) on BBB integrity and neuroinflammation in a controlled, human-relevant context^80^,^81^. To accelerate clinical translation, it is urgently necessary to establish an interdisciplinary technology maturity evaluation system that jointly considers biological validity, engineering feasibility, and clinical compliance. Currently, fewer than 20% of preclinical studies on microbiota-based AD interventions simultaneously meet standards for organoid validation, targeted delivery, and large-scale production^34^,^48^. Systems biology approaches have emerged as powerful tools to decipher the complex, multi-scale interactions between gut microbiota and AD pathology^4^,^47^. Constraint-based metabolic modeling, such as the COMETS platform, has been successfully applied to simulate microbial metabolic complementarity in AD mouse models, revealing that the depletion of butyrate synthesis pathways (the mechanistic consequences of which are elaborated in Section 5.2) creates a pro-inflammatory metabolic niche that accelerates amyloid pathology^47^,^77^. Moreover, genome-scale metabolic models (GEMs) of host-microbiota interactions have identified key metabolite exchange nodes, including acetate, succinate, and tryptophan derivatives, that modulate microglial activation states and tau phosphorylation kinetics^47^,^51^. Dynamic network modeling incorporating longitudinal multi-omics data has further shown that the C/EBPβ-AEP signaling pathway acts as a critical control point, where microbial-derived LPS and SCFAs exert opposing regulatory effects, providing a quantitative framework for predicting therapeutic responses to microbiota-targeted interventions^20^,^31^. It is urgently necessary to establish an interdisciplinary technology maturity evaluation system (such as a three-dimensional evaluation model of biological verification-engineering implementation-clinical compliance), to accelerate technology transformation (Table 2). In the development of microbiota regulation therapies, significant gaps remain between preclinical findings and clinical application. Current challenges include insufficient standardization of intervention protocols, limited long-term safety data, and a lack of validated biomarkers for monitoring treatment response. For example, although machine learning and network analysis have been incorporated into the study design of personalized dietary supplements for AD (e.g., a registered clinical trial, NCT06199193), these AI applications are currently limited to data integration for supplement formulation rather than direct therapeutic decision-making. While the integration of artificial intelligence holds conceptual promise for personalized treatment planning, such applications remain at the hypothesis-generating stage and require extensive prospective validation before clinical implementation (Table 3).

Artificial intelligence (AI) has become an indispensable tool for integrating heterogeneous multi-omics data in AD research. Deep learning architectures, particularly graph neural networks (GNNs) and transformer-based models, have been successfully applied to fuse gut metagenomic, metabolomic, and host proteomic data, enabling the identification of microbiota-derived metabolite panels that accurately discriminate AD patients from healthy controls (AUC > 0.90). Furthermore, causal inference algorithms (e.g., MR) combined with machine learning have established that specific gut microbial taxa, such as Bifidobacterium and Ruminococcus, exert causal effects on AD risk, independently of APOE ε4 genotype. AI-driven multimodal data integration also facilitates the development of personalized microbiota-targeted interventions by predicting individual responses to probiotics, prebiotics, or FMT based on baseline microbiome and metabolome profiles.

### 8.4 Critical appraisal of translational challenges, safety concerns, and standardization issues in microbiota-based AD therapies

At the specific intervention level, several critical barriers must be addressed before clinical translation of microbiota-based therapies. These intervention-specific challenges including safety concerns, formulation stability, and dosage optimization are discussed below. A broader appraisal of general limitations across the entire gut-brain axis field, including causality inference and methodological standardization, is consolidated in Section 10. Despite the promising potential of gut microbiota-targeted interventions for Alzheimer's disease (AD), several critical barriers must be addressed before clinical translation^15^,^32^,^85^. First, translational challenges arise from the inherent complexity of the gut-brain axis^3^,^34^,^86^. Preclinical studies predominantly use germ-free or transgenic mouse models (e.g., 5xFAD, APP/PS1), which do not fully recapitulate the aged, comorbid, and polymedicated human AD population^13^,^46^. Moreover, the temporal dynamics of microbiota composition (influenced by diet, antibiotics, and lifestyle) make it difficult to achieve sustained engraftment of exogenous probiotics or transplanted fecal microbiota^55^,^60^. A recent systematic review highlighted that less than 15% of microbiome-based interventions in animal models have progressed to phase I/II trials, primarily due to poor reproducibility across cohorts and lack of dose-response data^15^,^34^.

Second, safety concerns (beyond the causal and mechanistic limitations of FMT discussed in Section 4.2) remain underexplored. While FMT has shown benefits in some AD models, cases of FMT-transmitted multidrug-resistant infections and unexpected metabolic shifts have been documented in other indications^48^,^61^. Engineered probiotics (e.g., CRISPR-edited Lactobacillus) carry risks of horizontal gene transfer to commensal pathogens, potentially disseminating antibiotic resistance or pro-inflammatory genetic elements^72^,^79^. Long-term modulation of the gut microbiota may also disrupt the ecological resilience of the intestinal ecosystem, leading to opportunistic infections (e.g., Clostridioides difficile overgrowth) or aberrant immune activation, particularly in elderly AD patients with compromised mucosal barriers^60^,^61^. Furthermore, the use of postbiotics (e.g., butyrate, tryptophan metabolites) at supraphysiological doses raises concerns about off-target effects on hepatic metabolism and systemic inflammation^21^,^59^.

Third, standardization issues plague both research and clinical domains^13^,^56^. Current multi-omics studies exhibit substantial variability in sample collection (stool vs. mucosal biopsy), storage conditions (ambient vs. -80°C), DNA extraction kits, 16S rRNA primer sets, and bioinformatics pipelines (e.g., QIIME2 vs. mothur), leading to contradictory findings on which bacterial taxa are associated with AD^13^,^56^. An international ring-trial revealed that inter-laboratory reproducibility of gut microbiota composition analysis is only 47-62% for the same fecal samples^56^. For clinical applications, no consensus exists on the composition, viability, or dosing of “probiotic consortia” for AD; similarly, FMT lacks standardized donor screening protocols, treatment regimens, and outcome measures^36^,^48^. The absence of validated potency assays and stability tests for live biotherapeutic products (LBPs) further impedes regulatory approval by agencies such as the FDA or EMA^34^,^48^.

Taken together, addressing these translational, safety, and standardization gaps requires a coordinated effort involving preclinical model refinement, longitudinal human cohort studies with repeated sampling, and the establishment of international guidelines, such as those proposed by the International Microbiome Centre for FMT standardization^48^,^87^. Without such measures, the field risks replicating the cycle of oversimplified mechanisms and non-reproducible clinical results that have plagued prior AD therapeutic development^15^,^39^.

## 9. Critical Appraisal and Limitations of Current Evidence

Despite the substantial progress discussed above, several critical limitations constrain the interpretation and translational potential of current gut-brain axis research in Alzheimer's disease (AD). First, the vast majority of mechanistic insights, particularly those involving the C/EBPβ-AEP pathway, specific microbial metabolites (e.g., butyrate, quinolinic acid), and strain-specific bacterial effects, are derived from preclinical animal models (e.g., APP/PS1, 5xFAD, 3xTg mice). While invaluable for hypothesis generation, these models do not fully recapitulate the complex, aged, and multi-morbid human AD pathophysiology, and findings from murine systems cannot be directly extrapolated to humans without rigorous clinical validation. Second, human clinical evidence remains predominantly cross-sectional and correlational. Although robust associations between gut dysbiosis and AD biomarkers have been established, these study designs cannot definitively establish causality or distinguish whether microbial alterations drive neurodegeneration or reflect secondary consequences of the disease process, dietary changes, or medication use. Third, substantial methodological heterogeneity across studies including variations in fecal sampling protocols, DNA extraction kits, 16S rRNA variable regions, sequencing platforms, and bioinformatics pipelines undermines cross-cohort reproducibility and has hindered the identification of a universally reliable microbial signature for AD. Fourth, the field currently lacks large-scale, long-term, randomized controlled trials (RCTs) that employ AD-relevant primary endpoints (e.g., change in CSF p-tau/Aβ42 ratio, amyloid PET standardized uptake value ratio, or plasma phosphorylated tau) to rigorously evaluate the efficacy and safety of microbiota-based interventions such as probiotics, prebiotics, postbiotics, or FMT. These collective limitations highlight the need for cautious interpretation of current findings and underscore the critical priority areas for future mechanistic and translational research.

## 10. Summary and Prospects

### 10.1 Literature retrieval and review methodology

Given the broad and interdisciplinary scope of this review, we conducted a comprehensive narrative (integrative) review rather than a systematic review or meta-analysis. This approach is appropriate for synthesizing heterogeneous evidence spanning molecular biology, microbiology, neuroscience, and computational medicine to construct a conceptual framework for the gut-brain axis in AD^15^,^39^.

Search strategy and databases: We systematically searched the following electronic databases for peer-reviewed literature published up to March 1, 2025: PubMed/MEDLINE, Web of Science Core Collection, Scopus, and the Cochrane Library. The search strategy combined terms related to three core concepts: (1) Alzheimer's disease (e.g., "Alzheimer's disease", "AD", "amyloid-beta", "tau", "neurodegeneration"); (2) gut-brain axis and gut microbiota (e.g., "gut-brain axis", "gut microbiota", "intestinal microbiome", "dysbiosis", "probiotics", "postbiotics"); and (3) metabolism and interdisciplinary approaches (e.g., "metabolomics", "multi-omics", "SCFAs", "tryptophan metabolism", "system biology"). Boolean operators (AND, OR) were used to combine search terms. The complete search string for PubMed is provided in Search strategy and databases. Additional relevant articles were identified by manual screening of reference lists from included studies and key review articles.

Inclusion and exclusion criteria: Included studies were required to: (1) investigate the molecular mechanisms linking gut microbiota, the gut-brain axis, and AD pathology; (2) present original data from animal models, human clinical cohorts, or multi-omics analyses (metagenomics, metabolomics, transcriptomics, proteomics); (3) be published in peer-reviewed English-language journals; and (4) provide sufficient methodological detail for data extraction. Exclusion criteria were: (1) studies focusing exclusively on peripheral diseases without relevance to neurodegeneration or cognition; (2) conference abstracts, editorials, or opinion pieces without original data; (3) studies involving only in vitro cell cultures without in vivo validation; and (4) duplicate publications or studies with insufficient data quality as judged by consensus.

**Figure 6:**
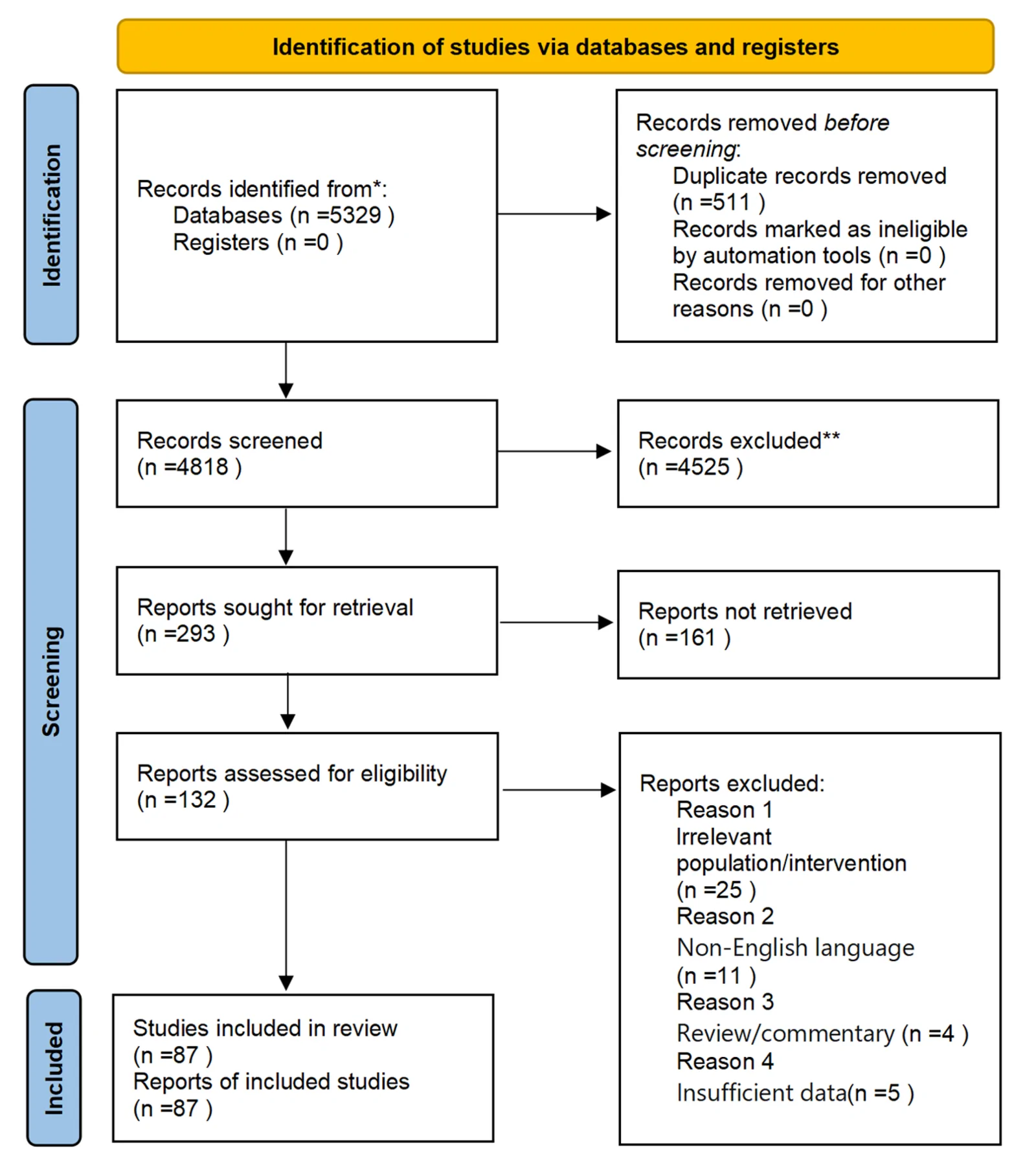
PRISMA flow diagram of the literature search and study selection process for this review. Only searches of databases and registers were included. *Consider, if feasible to do so, reporting the number of records identified from each database or register searched (rather than the total number across all databases/registers). **If automation tools were used, indicate how many records were excluded by a human and how many were excluded by automation tools. *From*: Page MJ, McKenzie JE, Bossuyt PM, Boutron I, Hoffmann TC, Mulrow CD, et al. The Prisma 2020 statement: an updated guideline for reporting systematic reviews. BMJ 2021;372:n71. doi: 10.1136/bmj.n71. For more information, visit: http://www.prisma-statement.org/.

Study selection and data synthesis: Two independent reviewers (P.L. and M.L.L.) screened titles, abstracts, and subsequently full texts against the eligibility criteria. Disagreements were resolved through discussion or arbitration by a third senior reviewer (Y.S.). The inter-rater agreement was high (Kappa = 0.91). Data were extracted into a standardized form covering study design, model system (human/animal), microbiota-related interventions or measurements, metabolic pathways, key molecular findings, and clinical outcomes. The extracted evidence was synthesized thematically according to the following conceptual domains: (1) causal associations between gut dysbiosis and AD pathology; (2) metabolic reprogramming mechanisms; (3) multi-omics integration findings; and (4) interdisciplinary intervention strategies. A PRISMA-compliant flow diagram outlining the study selection process is presented (Fig. 6).

Limitations: As a narrative review, this study does not include formal risk of bias assessment or quantitative meta-analyses. However, we have prioritized findings from well-controlled animal studies, replicated clinical observations, and mechanistic validations to ensure scientific rigor.

The preceding section (Section 9) has critically appraised the major limitations of current evidence in this field. Building upon these considerations, the following prospects outline key priorities and directions for future research. In recent years, substantial progress has been made in understanding the potential role of the gut-brain axis in AD pathophysiology, particularly through preclinical models and multi-omics approaches^28^,^29^. A growing body of evidence suggests that gut microbiota dysbiosis may influence the core pathology of AD through metabolic reprogramming, immunoinflammatory cascades, and molecular signaling pathways^1^,^64^. However, it is important to note that most of these mechanistic findings are derived from animal studies, and their direct relevance to human AD remains to be fully validated^2^,^15^. The deep integration of multi-omics technologies (such as the combined analysis of metagenomics, metabolomics, and spatial transcriptomics) has clarified the key molecular associations between microbiota metabolites (such as SCFAs and secondary bile acids) and host neurodegenerative diseases. Interdisciplinary research (such as the integration of microbiomics and artificial intelligence) has further promoted the development of microbiota-targeted intervention strategies, including the screening of probiotics, the optimization of postbiotic delivery systems, and the design of individualized microbiota transplantation programs^32^,^33^ (such as insufficient temporal resolution and lack of spatial localization) limits dynamic correlation analysis. In the future, it is necessary to combine germ-free animal models with longitudinal cohort studies, integrate single-cell spatiotemporal omics technologies, and verify the causal contributions of specific strains or metabolites to AD pathology through machine learning algorithms (such as causal inference models). Unaddressed issues regarding safety and standardization. Compared with the technical standardization of FMT such as operational specifications, the safety of long-term gut microbiota intervention in elderly patients with Alzheimer's disease (AD) is still severely understudied. Recent evidence has demonstrated that even supplementation with single-strain probiotics may lead to bacteremia in immunocompromised populations. Additionally, FMT has been linked to fatal transmission of extended-spectrum beta-lactamase (ESBL)-producing *Escherichia coli*. Furthermore, the absence of uniform reference materials for microbiome detection, including spike-in controls and universal reference specimens, greatly impedes comparative analysis across different studies. Hence, future clinical trials are required to implement full-scale safety monitoring measures, which cover metagenomic detection of antimicrobial resistance genes and long-term dynamic observation of host inflammatory biomarkers. It is urgent to establish an interdisciplinary technology verification system and develop unified operation protocols and efficacy evaluation standards. Beyond the scientific challenges, technical standardization and the gap between preclinical models and clinical practice represent major translational bottlenecks. Significant heterogeneity exists across studies in fecal sample handling (room-temperature vs. immediate freezing), DNA extraction methods (bead-beating parameters), 16S rRNA variable regions (V3-V4 vs. V4-V5), and bioinformatics pipelines (QIIME2 vs. DADA2 vs. UPARSE), leading to poor reproducibility. Although the International Human Microbiome Standards (IHMS) has issued guidelines, they are not yet widely adopted in AD research. Furthermore, commonly used AD mouse models (5xFAD, APP/PS1) have gut microbiota compositions that differ markedly from humans (e.g., much higher Lactobacillus abundance in mice). Therefore, humanized microbiota mice combined with AD-relevant genetic backgrounds should become the gold standard for preclinical validation. Future efforts should establish unified data reporting standards (e.g., MIxS-BE) for AD microbiome studies and validate cross-population applicability of candidate biomarkers through multicenter, multi-ethnic longitudinal cohorts such as the MIND-AD consortium. The need to optimize the interdisciplinary collaboration mechanism, disciplinary barriers and the dispersion of resources lead to low efficiency in multidisciplinary collaborative design. Solutions include constructing open data sharing platforms (such as the AD microbiome database), improving the training system for compound talents (such as cross-disciplinary courses in computational biology and clinical medicine), and formulating an intellectual property allocation framework across institutions.

Future translational priorities should be stratified by feasibility. In the near term, harmonizing multi-omics methodologies and establishing longitudinal cohorts with repeated fecal/plasma sampling are essential to distinguish causality from epiphenomenon. In the medium term, rigorously designed randomized controlled trials of well-characterized postbiotics (e.g., nano-encapsulated butyrate) and synbiotics, incorporating AD-relevant biomarker endpoints (e.g., CSF p-tau/Aβ42), are needed to define efficacy and safety profiles. In the longer term, advanced tools including CRISPR-engineered probiotics and AI-driven causal inference models may offer personalized modulation; however, substantial hurdles in strain stability, regulatory approval, and colonization efficiency must be overcome, and these approaches currently remain at the preclinical proof-of-concept stage.

## Figures and Tables

**Table 1:** Summary of key human clinical evidence for gut microbiota alterations in Alzheimer's disease.

Study Type / Evidence Level	Representative Findings	Key Limitations & Biases	Translational Gap & Implications
Cross-Sectional Case-Control Studies(Level III-IV)	• Consistent reduction in microbial α-diversity and butyrate-producing genera (*Faecalibacterium*, *Roseburia*)^10^,^11^,^17^.• Variable findings at the phylum level (e.g., inconsistent changes in *Bacteroidetes*), reflecting substantial inter-individual heterogeneity^13^,^15^.• Gut microbiota composition exhibits moderate accuracy (AUC ≈ 0.68) in discriminating preclinical AD from healthy controls^41^.	• Cannot establish causality: Dysbiosis may be a consequence of AD (e.g., driven by diet, lifestyle, or medications) rather than a cause^15^,^42^.• Confounding bias: Uncontrolled factors (polypharmacy, dietary patterns, comorbidities) strongly shape microbiota composition^42^.• Methodological heterogeneity: Lack of standardization in sampling, DNA extraction, sequencing, and bioinformatics across cohorts hinders meta-analyses^13^,^56^.	• Microbiota-derived signatures represent associative biomarkers and are not yet ready for clinical diagnostic use.• Findings are hypothesis-generating, identifying candidate taxa (e.g., *Bacteroides fragilis*, *Clostridium* spp.) for mechanistic validation in animal models^19^,^45^.
Prospective Longitudinal Cohort Studies(Level II-III)	• Lower baseline abundance of *Bifidobacterium* and higher levels of *Enterobacteriaceae* are associated with an increased hazard of progression from MCI to AD dementia^43^,^44^.• Dynamic changes in circulating inflammatory markers (IL-6, CRP) correlate with clinical AD stages^1^,^22^.	• Short follow-up periods (typically 2-5 years) fail to capture the decades-long prodromal phase of AD^15^,^39^.• Residual confounding: Time-varying confounders such as diet and medication changes are difficult to fully adjust for.• Single-timepoint measurements cannot capture intra-individual temporal variability of the gut microbiota^13^,^55^.	• Provides stronger temporal support for a potential causal direction from dysbiosis to neurodegeneration.• Underscores the need for long-term, multi-timepoint sampling cohorts with deep clinical phenotyping.
Metabolomics & Inflammatory Marker Studies(Level III)	• Reduced circulating SCFAs (particularly butyrate) correlate with higher cerebral Aβ burden^12^,^49^.• Shift in tryptophan metabolism toward the neurotoxic kynurenine pathway (elevated K/T ratio)^9^,^50^.• Elevated serum LPS and LBP (markers of microbial translocation) are associated with severity of cognitive impairment^1^,^11^.	• Peripheral metabolic interference: Metabolite levels are influenced by host factors (renal function, diet, hepatic metabolism) independent of the gut microbiota^42^,^51^.• Modest effect sizes and substantial overlap between diagnostic groups limit diagnostic utility^15^,^49^.• Lack of disease specificity: Whether these changes are specific to AD versus other dementias remains largely unexplored^2^,^39^.	• Corroborates preclinical mechanistic pathways in human populations.• Indicates that peripheral metabolites cannot serve as reliable standalone biomarkers without accounting for systemic host physiology.
Human-to-Animal FMT Studies(Level II, experimental)	• Transplantation of AD-patient microbiota into germ-free mice induces AD-like pathologies: impaired hippocampal neurogenesis, cognitive deficits, and increased Aβ deposition^30^,^31^,^45^.	• Cross-species translatability limitations: Murine and human gut ecosystems differ substantially in composition, metabolism, and immune architecture^13^,^46^.• Artificial dosing: The quantity of transplanted material far exceeds physiological human exposure scenarios^48^.• Use of young healthy recipients may overestimate effect sizes relative to aged, genetically susceptible hosts^2^,^46^.	• Provides the strongest experimental evidence for a pro-pathogenic property of AD-associated microbiota.• Does not constitute clinical evidence; findings must be validated in human intervention trials.
Interventional Clinical Trials (Probiotics / Prebiotics / FMT)(Level I-II)	• Some small-scale trials report improvements in cognitive scores and changes in inflammatory markers in patients with AD or MCI^57^,^58^.• Preliminary evidence suggests FMT may improve cognitive outcomes in AD patients with comorbid conditions^36^.	• Small sample sizes and high dropout rates reduce statistical power^57^,^58^.• Heterogeneous intervention protocols: Optimal formulation, dosing, and patient stratification remain undefined^59^,^60^.• Lack of robust AD-specific endpoints: Most trials use cognitive scales as primary outcomes; few incorporate CSF biomarkers or amyloid PET^32^,^52^.• Safety concerns: Risk of bacteremia with probiotics and pathogen transmission with FMT in vulnerable populations^48^,^61^.	• Fundamental translational gap: No large-scale, double-blind RCT has yet demonstrated that gut microbiota modification slows cognitive decline or alters core AD biomarkers in patients.• Rigorous RCTs with AD-relevant primary endpoints are urgently needed to translate preclinical promise into clinical practice.

**Table 2:** Comparative analysis of conflicting findings in gut microbiota-Alzheimer's disease (AD) research: mechanistic differences, limitations and translational implications.

Research Topic	Key Divergent Findings	Comparative Interpretation	Major Limitations in Current Evidence	Translational Relevance to AD Pathology & Microbiota-Targeted Interventions
AD pathological mechanisms & gut microbiota association	Some studies report decreased Bacteroidetes abundance in AD, while others find no significant change or even an increase^10^,^11^,^15^,^17^.	Discrepancies likely arise from differences in disease stage (early vs. late AD), sample source (fecal vs. mucosal), and geographic dietary patterns. Bacteroidetes may change dynamically with neuroinflammation severity^15^,^42^.	Cross-sectional designs; lack of longitudinal tracking; confounding by medication (e.g., cholinesterase inhibitors) and diet^15^,^42^.	Suggests that microbiota-based biomarkers must be stage-specific. Interventions should consider baseline Bacteroidetes levels to predict response to prebiotics/probiotics^15^,^17^.
Role of gut microbiota metabolites (SCFAs, tryptophan, LPS)	Butyrate is consistently reduced in AD models, but its effect on tau pathology is model-dependent: protective in 3xTg but not in 5xFAD mice^7^,^23^,^46^.	Genetic background modulates butyrate's HDAC-inhibitory activity and microglial response. Tryptophan-kynurenine shift is robust across models, but quinolinic acid levels correlate with Aβ only in aged mice^21^,^50^.	Most metabolite data from fecal or serum samples, not from CNS interstitial fluid; causal direction unclear^7^,^26^.	Butyrate supplementation may only benefit AD subtypes with intact HDAC sensitivity. Personalized metabolite profiling needed before intervention^21^,^33^.
Multi-omics applications (metagenomics, metabolomics, spatial transcriptomics)	Some studies show strong correlation between Clostridium abundance and brain 4-HNE (lipid peroxidation) , but others find no significant link in human cohorts^25^,^28^.	Clostridium species are functionally diverse: some produce butyrate, others generate LPS. The positive correlation may be driven by pro-inflammatory species, not the genus as a whole^13^,^14^,^18^.	Lack of strain-resolution sequencing; multi-omics integration remains descriptive, not predictive; no spatial-temporal dynamics^15^,^73^.	Strain-specific targeting (e.g., C. butyricum vs. C. perfringens) is essential. AI-driven metagenomic classifiers must move to functional gene-level analysis^14^,^74^.
Gut microbiota-targeted intervention strategies (probiotics, FMT, postbiotics)	FMT from AD patients to germ-free mice consistently induces cognitive deficits, but FMT from healthy donors to AD mice only partially restores cognition in some studies^30^,^31^,^36^.	Efficacy of healthy FMT depends on donor microbiota diversity and recipient's baseline dysbiosis severity. Some beneficial strains fail to engraft due to niche competition^32^,^60^.	Short follow-up periods; no standardization of FMT preparation or dosing; lack of sham-controlled human trials^48^,^87^.	Success requires pre-FMT conditioning (diet, antibiotics) and post-FMT monitoring of engraftment. Engineered probiotics with colonization factors may improve persistence^32^,^72^.
Critical appraisal of evidence strength	Preclinical causal claims vs. human correlational data	Animal FMT models show strong effects, but human RCTs lack robust AD-primary endpoints	Over-reliance on germ-free models; absence of longitudinal human mechanistic validation	Strain-specific and metabolite concentration-dependent effects must guide future trial design

**Table 3:** Clinical translation challenges in gut microbiota-Alzheimer's disease (AD) research: root cause analysis, solution feasibility and future directions.

Clinical Translation Challenge	Root Cause / Mechanism	Proposed Solution	Feasibility Assessment	Link to AD Pathology & Microbiota Intervention
Peripheral metabolic interference	Microbiota-derived metabolites in blood are diluted and confounded by host metabolism (e.g., liver, adipose tissue)^26^,^49^.	Identify brain-derived or CSF-specific metabolite signatures (e.g., quinolinic acid, 5-HIAA) that cross the BBB^26^,^50^.	Moderately feasible: requires CSF collection, limiting scalability. Alternative: develop CNS-enriched exosome biomarkers from blood^26^,^37^.	Enables early detection of tryptophan-kynurenine axis dysregulation before cognitive decline^41^,^50^.
Lack of early AD-specific microbial biomarkers	Most studies recruit mild-to-moderate AD patients; preclinical (MCI) cohorts are small and lack multi-omics follow-up^12^,^15^,^41^.	Establish prospective longitudinal cohorts with MCI individuals, collecting fecal/plasma/CSF at 6-month intervals^43^,^55^.	High feasibility but requires large funding and multi-center collaboration. Pilot studies ongoing (e.g., ADNI-Microbiome)^17^,^55^.	Early detection allows preventive interventions (e.g., postbiotics) to delay Aβ aggregation and tau spreading^33^,^59^.
Animal model translation obstacles	Germ-free or antibiotic-treated AD models do not fully recapitulate human age-related dysbiosis; human microbiota transplant models have short colonization windows^30^,^46^.	Use humanized gnotobiotic mice colonized with AD-patient microbiota aged in situ, combined with high-fat or Western diet to mimic human lifestyle^19^,^46^.	High feasibility for mechanistic studies; cost and time remain barriers. New "avatar mouse" platforms are emerging^30^,^46^.	Validates causality of specific bacterial strains in driving C/EBPβ-AEP pathway activation^19^,^31^.
Multi-omics data integration difficulty	Data heterogeneity (batch effects, platform differences) and lack of interpretable AI models lead to spurious correlations^15^,^56^.	Develop knowledge graph-based integration (e.g., microbiome-metabolite-protein interaction networks) with attention-based deep learning^73^,^74^.	Moderate feasibility: requires standardized ontologies and large training datasets. The iNAP 2.0 platform offers a starting point^73^.	Uncovers nonlinear interactions (e.g., bile acids × ApoE4 × Aβ) that single-omics approaches miss^16^,^64^,^68^.
Intervention measure limitations (probiotic instability, FMT variability)	Live probiotics lose viability during storage and passage through GI tract; FMT donors vary in microbial composition (see Section 4.2 for the interpretive caveats of FMT studies)^32^,^48^,^60^.	Use next-generation postbiotics (encapsulated butyrate, nano-formulated 5-HT precursors) or engineered probiotics with sporulation capacity^33^,^59^,^72^.	High feasibility: several postbiotics are already in Phase II trials for AD (e.g., sodium butyrate enemas)^21^,^33^.	Directly targets metabolite deficiencies without requiring live bacterial engraftment, reducing safety concerns in immunocompromised elderly^61^,^87^.

## Data Availability

All data included in this review article are derived from previously published studies and are freely available in the respective databases or journals. No additional raw data were generated for this review.
